# ExLightNet: A Lightweight and Explainable 1D-CNN for Real-Time Intrusion Detection in Resource-Constrained IoT Sensor Networks

**DOI:** 10.3390/s26217023

**Published:** 2026-11-06

**Authors:** Saman Iftikhar, Ammar Rafiq, Syed Rizwan Hassan

**Affiliations:** 1 Faculty of Computer Studies, Arab Open University, Riyadh 84901, Saudi Arabia; 2 Department of Software Engineering, National University of Computer and Emerging Sciences, Islamabad, Chiniot-Faisalabad Campus, Chiniot 35400, Pakistan; ammar.rafiq@nu.edu.pk; 3 Department of Computer Engineering, Gachon University, Seongnam 13120, Republic of Korea

**Keywords:** IoT security, IoMT, intrusion detection system, lightweight deep learning, explainable artificial intelligence, SHAP, model quantisation, edge computing, network sensors

## Abstract

Internet of Things (IoT) and Internet of Medical Things (IoMT) sensor deployments are pushing security decision-making from centralised servers towards resource-constrained edge gateways, where heavyweight deep learning detectors exceed the available compute, memory, and latency budgets, and where opaque alarms erode operator trust. This paper proposes ExLightNet, a lightweight one-dimensional convolutional neural network that couples depthwise-separable convolutions and squeeze-and-excitation channel attention with a tempered class-balanced training recipe (focal loss plus $\sqrt{f^{-1}}$ sampling) and per-channel INT8 weight quantisation, and attaches SHAP attributions directly to the deployed model. We evaluate the framework on the public RT-IoT2022 benchmark of 123,117 real bidirectional flows from smart-home and sensor IoT devices spanning nine attack behaviours and three normal device classes, an environment representative of the auxiliary sensing layer of home-based care settings. ExLightNet attains an attack-class recall (sensitivity) of 0.9976, missing only 40 of 16,592 attack flows, with macro-F1 0.9860 and AUC 0.9985 for binary attack detection (the 99.49% accuracy is retained for comparability with prior work, but accuracy is a misleading headline metric under the severe class imbalance of network traffic), and a macro-recall of 0.863 (97.70% accuracy, macro-F1 0.8621) across twelve fine-grained classes, surpassing a 4.5× larger standard CNN on the binary task while remaining within 0.0021 macro-F1 of Random Forest. Post-quantisation, the model shrinks from 162 KB to 37 KB (4.4×) with a macro-F1 drop of at most 0.007, sustaining 0.50 ms single-flow CPU latency, 66× faster than an accuracy-equivalent Random Forest. On a Raspberry Pi 4 gateway, the INT8 model sustains 2197 flow decisions per second across four Cortex-A72 cores, 21.4× faster than an accuracy-equivalent Random Forest, confirming the deployment profile on measured gateway-class hardware. SHAP analysis shows that forward payload statistics, backward header-size extremes, and packet-rate features dominate decisions, yielding operator-readable rationales.

## 1. Introduction

The sensor layer of the Internet of Things (IoT) is the sensory nervous system of modern digital infrastructure, bridging billions of environmental, physiological and operational sensors to applications ranging from smart-home automation to continuous patient monitoring. In the healthcare sector, the Internet of Medical Things (IoMT) has now enabled remote patient monitoring, smart wards, wearable telemetry, and hospital asset tracking, and the number of publications on the security issues of IoMT has increased over the years [1,2]. Importantly for this paper, the same commercial off-the-shelf consumer devices, voice assistants, telemetry nodes, smart lighting and Message Queuing Telemetry Transport (MQTT) brokers populate the auxiliary sensing layer of such deployments, as they do smart homes providing care-at-home services. As such, detection frameworks validated on real consumer-IoT traffic faced with the same amount of devices and traffic types as at the IoMT edge, but direct validation on clinical-protocol traffic is still pending (Section 5.3 and Section 5.9). The downside of this expansion, however, is that each new endpoint adds to the network’s attack surface, and constrained devices often come with well-known credentials and unpatched firmware and with communication channels that are unencrypted. The attacks on the IoT are reported to have been consistently increasing over the last few years: botnet recruitment, Distributed Denial-of-service (DDoS) attacks, protocol-level reconnaissance and brute-force attacks [3,4]. If the monitored devices contain patient information or control clinical equipment, successful attack will not only be a privacy incident, but also a potential safety hazard, making the stakes higher on network edge attack detection systems [5].

In response, machine learning (ML) based Intrusion Detection Systems (IDSs) have been growing more accurate with new transformer-style and ensemble architectures achieving outstanding performance on large public datasets [6,7]. But the architectures that perform best on accuracy are typically those that are the least appropriate to deploy: A model with millions of parameters, or an ensemble of a hundred or more decision trees can’t fit in the memory budget of a gateway-class device and can’t provide the deterministic sub-millisecond response that is required for inline traffic policing. To solve the aforementioned problem, a parallel research direction is being pursued to develop lightweight models for IDS such as knowledge-distilled detector, pruned networks and compact gradient-boosting variants [8,9]. In related IoT research, for example, a quantised AE was shown to compress the memory required to detect the objects on a Raspberry-Pi-like device without compromising the detection accuracy, obtaining important results for a real-world problem in quantisation [10].

Less talked about, there’s opacity, too. Deep models are often viewed by network operators and even more so by clinical personnel as black box alerts without explanations [11]. If an alarm is issued for no apparent reason in an IoMT environment, the alarm is either ignored or an expensive manual investigation is conducted, thereby reducing the trust in the IoMT system. Several Explainable Artificial Intelligence (XAI) techniques have therefore been suggested to add human-readable feature attributions to the outcomes of IDS decisions, including SHapley Additive exPlanations (SHAP) and Local Interpretable Model-agnostic Explanations (LIME) [12,13,14]. A combination of all three of the desiderata, however, in a validated framework is still scarce in the literature: (i) competitive accuracy for multi-class detection on a real traffic benchmark, (ii) an architectural and quantisation story that works within constraints on the edge, and (iii) a principled explanation layer that maps model decisions to actionable evidence for operators. The surveys on the study of XAI-empowered IoT IDSs explicitly request studies that combine interpretability and deployability, instead of interpretability being the afterthought [11,15]. Existing studies usually focus on one or two of these pillars.

This paper closes that gap with ExLightNet, a lightweight and explainable 1D-CNN framework designed for inline deployment on IoT and IoMT gateways. The framework converts Zeek-derived flow records from the public RT-IoT2022 benchmark [16] into per-flow decisions, selects forty discriminative features through mutual-information ranking, trains a depthwise-separable CNN with Squeeze-and-Excitation (SE) channel attention [17] using focal loss [18] combined with a tempered $\sqrt{f^{-1}}$ class-balanced sampler, and finally, compresses the trained model through per-channel INT8 weight quantisation. Every decision is accompanied by SHAP attributions [14] computed directly on the compact model, so that operators receive the same evidence trail they would expect from a much heavier system. We summarise the contributions as follows:

- A co-designed architecture and training recipe for edge IDS. We propose ExLightNet, a 35–38K-parameter 1D-CNN whose depthwise-separable blocks and dual global pooling head are matched to a focal-loss-plus-tempered-sampling recipe that stabilises learning under extreme class imbalance (an imbalance ratio of 3381:1 between the largest and rarest corpus classes, and 3313:1 within the training partition).
- A validated compression and efficiency profile. Per-channel INT8 weight quantisation shrinks the model from 162 KB to 37 KB with a macro-F1 penalty of at most 0.007, while the FP32 model already delivers 0.50 ms single-flow CPU latency, roughly 66× faster than an accuracy-equivalent Random Forest ensemble.
- An explainability layer grounded in the edge model itself. We provide global and local SHAP analyses of the deployed model, revealing that forward payload statistics, backward header-size extremes, and packet-rate features carry most of the discriminative evidence, and demonstrating that explanations remain faithful for the compact model.
- A comprehensive empirical study. Across binary and twelve-class tasks on RT-IoT2022, we benchmark ExLightNet against five external baselines (Std-CNN, BiLSTM, MLP, Random Forest, and XGBoost) plus a distillation variant of our own model, and report confusion matrices, ROC curves, per-class behaviour, and a five-way ablation covering SE attention, separable convolutions, sampling strategy, the feature budget, and distillation.

The rest of this paper is organised as follows. Related work is discussed in Section 2 where we review related work in IoMT security, IoT lightweight IDS and explainable detection. The dataset used, preprocessing pipeline, ExLightNet architecture, training recipe, compression strategy and explainability protocol are presented in Section 3. The results of the experiments are discussed in Section 4 and their implications, along with comparisons to the literature and limitations, are discussed in Section 5. In Section 6 conclusions are made and some future work is outlined.

## 2. Related Work

### 2.1. Security of IoT and IoMT Sensor Networks

The security field for healthcare-related IoT has grown very quickly. Ibrahim et al. [1] categorize attacks in smart healthcare systems, emphasizing that the different types of sensors and protocols increase the number of potential denial of service, spoofing, and eavesdropping attacks. Abbas et al. [2] review privacy-preserving learning for smart healthcare and identify that traffic that is centrally collected to be used for the detection, which is closer to the source of the data, is a disadvantage. Edge–cloud partitioning of healthcare analytics buffers the risk of traffic centralisation while reducing response latency further, all while maintaining privacy. As for the attack, systematic reviews of IoT botnet and DDoS campaigns [3] and of deep-learning countermeasures for IoT DDoS [4] confirm that volumetric attacks and protocol abuse attacks against consumer devices continue to be the primary operational stressors. Together, these studies support the framing of our work: detection needs to be done at the periphery of the sensor network, on commodity gateway hardware, and, importantly, needs to distinguish between benign device behaviour and a wide range of reconnaissance and intrusion techniques. Indeed, the latest detectors for IoMT traffic are designed to be protocol-aware, as in the case of attention-enhanced deep model of MQTT traffic proposed by Singhal and Kumar [5] for medical IoT deployment, where lightweight, protocol-sensitive detection is still an active need, not a solved issue, in 2026.

### 2.2. Lightweight Intrusion Detection for Edge Deployment

There is a growing literature that makes IDS optimizations for constrained execution environments. Hozouri et al. [19] and Ahanger et al. [20] perform a survey on machine-learning IPSs for IoT, and consistently make model footprint and inference latency as first class evaluation metrics in addition to accuracy. This finding is echoed by Bankó et al. [21] in a research-trend analysis, which shows parameter-efficient deep architectures are replacing monolithic models. Concrete examples can be found in Wang et al. [8], who present the self-knowledge distilled detector that compresses a teacher network into a compact student with the ability to detect multiple classes, and in Wu et al. [9], who introduce the curriculum knowledge-distillation framework, which schedules the difficulty of samples to achieve a better accuracy–size trade-off on IEEE IoT-Journal-class benchmarks. Both Wisanwanichthan and Thammawichai [22] and Ornob and Mtawa [23] propose a dual-branch deep network for IoT and UAV platforms under tight memory constraints, and show multiclass detection on microcontroller-class hardware, respectively. From an architectural perspective, our design’s depthwise-separable convolution is a popular primitive used by MobileNets [24] and has been the default option for resource-constrained inference, and Badhan [15] recently showed how to combine compact neuro-symbolic learners with interpretability for that very IoT edge scenario. Our contribution for this thread is motivated by two observations: One, most lightweight IDS studies optimise the model; Two, they propose quantisation or distillation as an optional extra, but we combine it with architectural efficiency and imbalance-aware training into one validated pipeline and report quantitative evidence of the deployment (latency, on disk size, and accuracy after compression) for every model in our comparison.

### 2.3. Explainable AI for Intrusion Detection

Explainability is no longer a philosophical bolt-on, it’s an operational component of security analytics. Gaspar et al. [12] systematically assess LIME and SHAP for multi-purpose IDSs, and demonstrate that flow-level features can be attributed reliably with both methods; SHAP has also been compared to LIME in a forensic context by Hermosilla et al. [25] where both shared some strengths, while SHAP provides more consistent guarantees through its axiomatic foundation [14]. Hulayyil et al. [13] present several evidence-based IoT datasets and suggest that explainable detection can help speed up the analyst triage workflow per feature. Varol and Karakaya [11] conducted the latest survey of XAI-based IDSs in IoT environments and found there were two open challenges: explanations are typically generated for large-sized cloud-side models, but not for the small-sized models deployed, and interpretability is rarely assessed together with behaviour changes induced by compression. Federated variants, like FedXAI [26] have been introduced to bring the benefits of explainability to decentralised training, and Kalakoti et al. demonstrate that attribution quality can be preserved under distributed optimisation. Both of our contributions directly address the above open problems: the SHAP layer is attached to the compact network whose computation graph is preserved verbatim under the Weight-only (W8) quantisation, rather than being attached to a separate heavyweight cloud surrogate; and we report the attribution landscape of the class of models deployed, which is dominated by packet-rate, header-size, and payload-size statistics.

### 2.4. Transformer- and Ensemble-Based Detectors

Transformer architectures have proven their value to IoT intrusion detection. Recent systems released in 2026, such as the transformer-based multi-class detector CyberRoBERTa [27] (small and language-model inspired), and the transformer of Hssayeni and Mahgoub [28] (zero day-oriented), have also shown high multi-class accuracy, continuing the trend of 2023 systems by Tseng et al. [6] which already reported high multiclass accuracy, with follow-up work done on mixed IoT corpora that validate this trend. Though not commonly used, ensemble methods are still formidable baselines for tabular flow features, particularly XGBoost [29] which is often leveraged as a reference detector for IoT benchmarks and is used in an optimised federated pipeline by Karunamurthy et al. [7]. These models are good at accuracy, but in their plain form they do not meet edge constraints: our model that consists of 400 gradient-boosted trees requires 9.4 MB, which is approximately 58× the size of ExLightNet. Compact transformer variants that accept fixed-length flow-feature vectors as input do exist though: the revision of our positioning is explicit about it, and we explicitly intend to make a controlled comparison under identical inputs as a future task.

### 2.5. Gap Analysis and Positioning

Table 1 summarises representative recent systems against five dimensions that matter for real IoT/IoMT adoption: architectural compactness, imbalance-aware training, post-training compression, built-in explainability, and evaluation on the real-time RT-IoT2022 benchmark. The comparison makes the gap visible. Quantisation-only work [10] validates compression but not multiclass discrimination or explanations; distillation frameworks [8,9] compress accurately but do not expose operator-facing evidence; XAI-centric studies [12,13] explain heavyweight models rather than deployable ones. To the best of our knowledge, no prior work couples a sub-40K-parameter neural detector, INT8 compression, and model-native SHAP explanations on real IoT traffic with a full efficiency audit.

## 3. Materials and Methods

**Figure 1:**
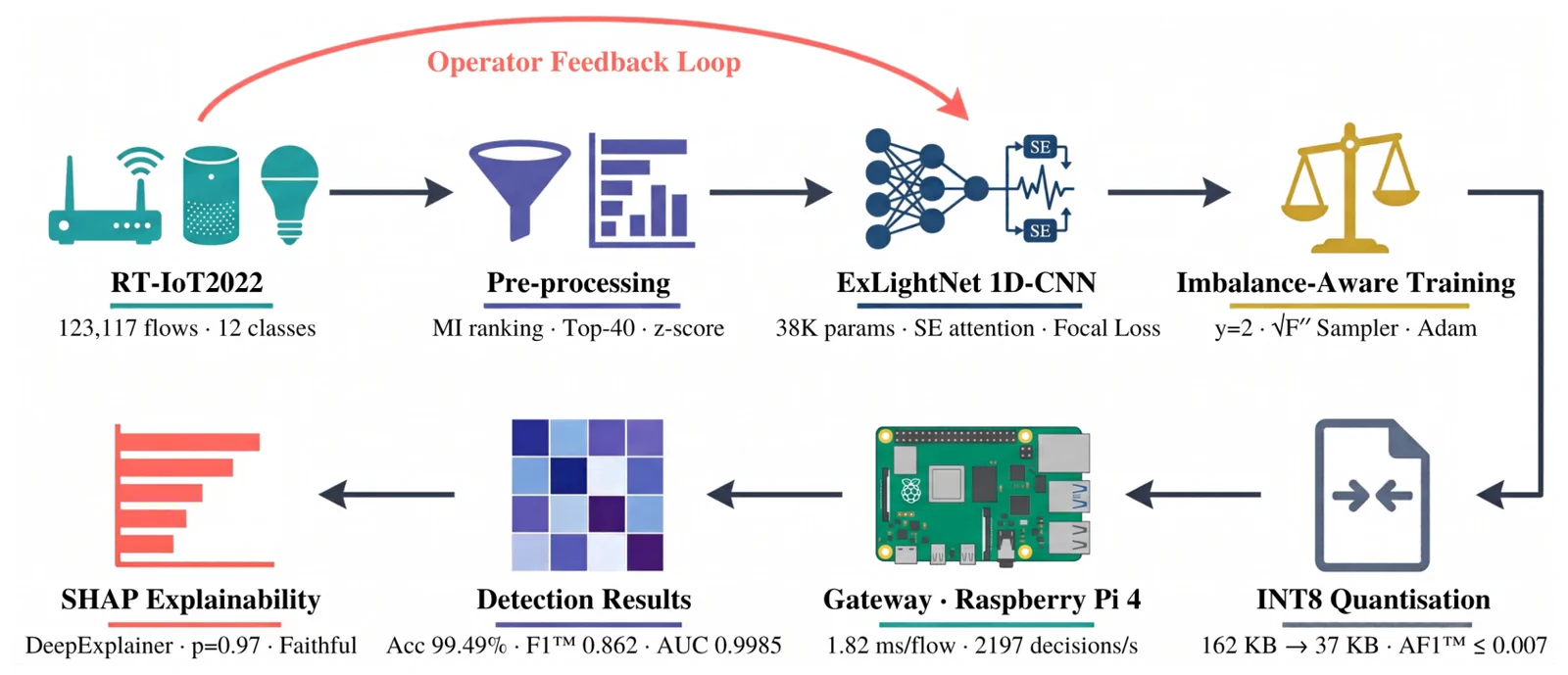
Overview of the proposed ExLightNet framework: data acquisition, pre-processing, edge model training with compression, and evaluation with SHAP-based explainability and an operator feedback loop.

Figure 1 presents an end-to-end view of the proposed framework. It comprises four coupled stages: (1) acquisition of real IoT traffic from the public RT-IoT2022 benchmark; (2) cleaning, encoding, mutual-information feature selection, and stratified partitioning; (3) imbalance-aware training of the ExLightNet edge model followed by per-channel INT8 quantisation; (4) deployment-oriented evaluation combined with SHAP-based explanation and an operator feedback loop. Each stage is described in the remainder of this section. Throughout, we assume a gateway-centric deployment model: ExLightNet runs once on the edge gateway that aggregates traffic from (potentially heterogeneous) sensor populations, rather than on each individual sensor node. The implications of device heterogeneity for a unified model and the mechanisms by which the framework adapts to heterogeneous hardware and local traffic profiles without per-device optimisation are analysed explicitly in Section 5.4.

### 3.1. Dataset: RT-IoT2022

We adopt the RT-IoT2022 dataset [16], a public real-time intrusion detection benchmark generated on a dedicated testbed of consumer IoT devices that is representative of the device population found in smart-home and assistive-health deployments, including Amazon Alexa speakers, Wipro smart bulbs, ThingSpeak telemetry nodes, and MQTT brokers [10]. Traffic was captured bidirectionally and summarised by Zeek into 83 statistical flow features covering packet counts, payload statistics, inter-arrival times (IAT), flag counts, bulk and subflow statistics, and TCP window behaviour. The corpus contains 123,117 labelled flows spanning nine attack behaviours, SYN-flood DoS (Hping), ARP poisoning, five NMAP reconnaissance variants (UDP, Xmas-tree, OS-detection, TCP-connect, and FIN scans), application-layer Slowloris DDoS, and Metasploit SSH brute-force, plus three benign device classes (ThingSpeak, MQTT publish, and Wipro bulb traffic). To the best of our knowledge, this is among the largest publicly available flow-level IoT corpora with device-identifiable normal traffic, and it has become a standard point of comparison for edge-oriented detectors [10]; complementary large-scale corpora such as TON_IoT combine telemetry and network services but lack the device-identifiable benign classes that RT-IoT2022 provides [30]. We deliberately retain all twelve classes rather than collapsing them, because the resulting 3381:1 imbalance between the largest class (DoS-SYN-Hping, 94,659 flows) and the rarest (NMAP-FIN-SCAN, 28 flows) reflects the operational reality that critical attack evidence is scarce.

**Figure 2:**
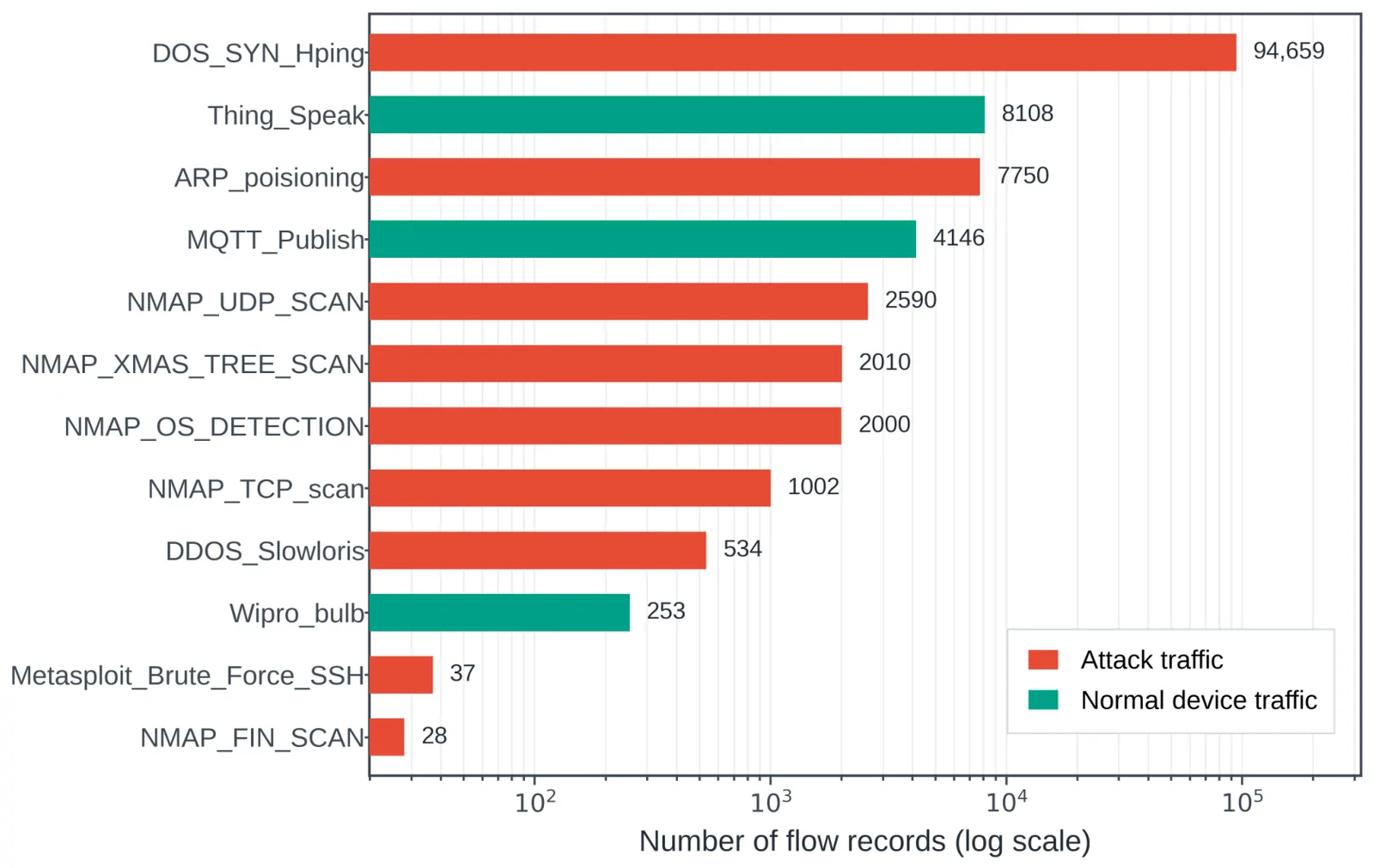
Class distribution of the RT-IoT2022 corpus (log scale). Attack classes (Red) span more than three orders of magnitude in volume, motivating imbalance-aware training. Class names follow the dataset’s original spelling.

Table 2 lists the per-class composition, and Figure 2 visualises the distribution. For the binary task, the three benign classes are mapped to a single normal label (12,507 flows, 10.2%), and the remaining nine classes form the attack label (110,610 flows, 89.8%). We use the UCI-repository release of the corpus (CC BY 4.0 licence) and report our stratified 70/15/15 train/validation/test split in Section 3.7. Because the released corpus is a flat per-flow CSV without capture-session identifiers, the split is a stratified random flow-level partition (seed 42); flows from the same attack run may therefore span partitions, and we treat this as a conservative setting that matches common practice on this benchmark [10]. The implications of this split design for generalisation are analysed explicitly in Section 3.8.

### 3.2. Pre-Processing and Feature Selection

The raw export contains an index column, two categorical protocol fields (proto, service), and 81 numeric statistics per flow. Our pipeline performs five steps. First, infinite and missing values produced by division-by-zero in flow statistics are repaired with per-feature medians. Second, the two categorical fields are one-hot encoded, expanding the candidate pool to 93 features, and the single constant feature (bwd_URG_flag_count) is pruned. Third, we rank features by Mutual Information (MI) with the binary attack-vs-normal target, computed only on the training partition to prevent selection leakage; the binary target is used because rare-class MI estimates from as few as 20–26 flows are unreliable, and Section 5.9 notes the resulting limitation for subclass separation; a comparison with a ranking computed against the twelve-class target is identified there as future work. Fourth, the top-40 features are retained at a budget chosen to balance discriminative power against the input width of the edge model; the ablation in Section 4.8 shows that halving this budget to 20 costs 0.26 macro-F1. Fifth, features are standardised with z-score statistics from the training partition. Table 3 groups the selected features by family; payload-size statistics (average, maximum, total, and standard deviation in each direction), subflow byte counts, and IAT extremes dominate the ranking, a pattern that reappears in the SHAP analysis of Section 4.6. Table 4 lists the selected features in MI-ranking order.

### 3.3. ExLightNet Architecture

**Figure 3:**
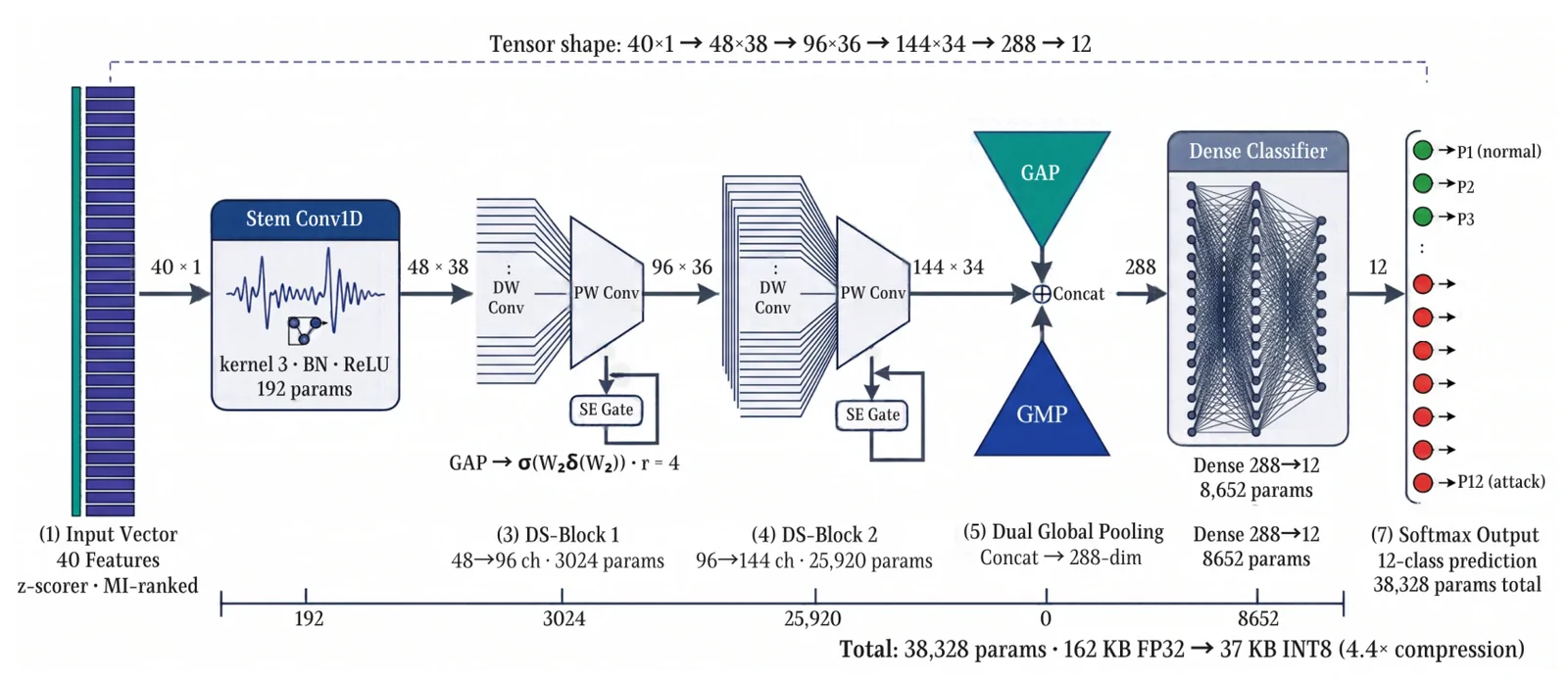
ExLightNet architecture. A 40-feature flow vector passes through a 1D stem convolution and two depthwise-separable blocks with SE channel attention; a dual global pooling head feeds a dense classifier. Parameter counts refer to the twelve-class variant.

Figure 3 shows the proposed network. A flow is treated as a length-40 one-dimensional signal in which position encodes feature identity. Positions follow the descending mutual-information ranking of Table 4: the highest-ranked feature occupies position 1, so the most discriminative statistics sit inside the dense cross-feature receptive field of the stem convolution, and adjacent positions group features of comparable global relevance rather than isolating any single measurement family at the signal tail. Because the ranking is computed once on the training partition and then frozen, the position-to-feature mapping is identical across training, validation, and every deployment site, which keeps the quantised artefact reproducible; the top-20-versus-top-40 ablation of Section 4.8 additionally shows that the leading MI positions carry most of the discriminative signal. The stem applies a conventional 3-tap Conv1D (1 → 48) with batch normalisation and ReLU, giving the network a first layer with full cross-feature receptive fields at negligible cost (192 parameters). Two depthwise-separable blocks follow. Each block pairs a depthwise 3-tap convolution (groups = channels) with a 1×1 pointwise projection that expands the representation 48→96→144, and inserts a Squeeze-and-Excitation (SE) gate [17] that re-weights channels according to a global descriptor:

(1) $$z=\sigma \;\left(W_{2}\;\delta \left(W_{1}\;\mathrm{GAP}\left(u\right)\right)\right),\;{\tilde{u}}_{c}=z_{c}\cdot u_{c},$$

where u is the block output, $W_{1}\;\in \;R^{C/4\times C}$ and $W_{2}\;\in \;R^{C\times C/4}$ form the excitation bottleneck, δ is ReLU, and σ is the sigmoid. The SE gate costs only $2C^{2}\;/4$ parameters per block yet lets the model emphasise discriminative feature channels (for example, the payload-variance and header-size channels that dominate the MI ranking), which our ablation quantifies at +0.066 macro-F1.

Depthwise separability is the key efficiency device: a standard convolution with kernel *k*, $C_{\mathrm{in}}$ input, and $C_{\mathrm{out}}$ output channels costs $k\;C_{\mathrm{in}}C_{\mathrm{out}}$ weights, whereas the separable equivalent costs $k\;C_{\mathrm{in}}+C_{\mathrm{in}}C_{\mathrm{out}}$, a 2.9× reduction for the widest block in ExLightNet (6.9× across the whole convolutional trunk) [24]. The head concatenates global average pooling (GAP) and global max pooling (GMP) over the 144 feature maps into a 288-dimensional embedding and applies a single dense layer to the class logits. The complete model has 35,438 parameters for the binary head and 38,328 for the twelve-class head (150 KB of FP32 weights; 162 KB on disk including serialisation overhead), versus 158,860 parameters (632 KB on disk) for our standard-convolution control with identical widths, i.e., a 4.1× structural reduction before any quantisation.

### 3.4. Imbalance-Aware Training

Two mechanisms address the 3381:1 class skew. The first is the focal loss [18],

(2) $$L_{\mathrm{FL}}=-\frac{1}{N}\sum_{i=1}^{N}\alpha_{y_{i}}\;{\left(1-p_{i,y_{i}}\right)}^{\gamma }\;\log p_{i,y_{i}},$$

with focusing parameter γ=2 and class-balanced weights α, which down-weights the gradient contribution of easy majority-class examples and concentrates capacity on hard rare-class flows. The second is a tempered class-balanced sampler: mini-batches are drawn with replacement, where the probability of class *c* is proportional to $f_{c}^{-1/2}$ ($f_{c}$ = class frequency). The square-root tempering deliberately avoids the pathology of fully balanced sampling, which would repeat each of the 20 training flows of the rarest class roughly 359× per epoch (versus roughly 30× under tempering) and destabilise batch normalisation; the multiclass ablation in Section 4.8 confirms that fully balanced sampling costs 0.081 macro-F1 relative to the tempered scheme.

All models are trained with Adam (learning rate $1.5\times 10^{-3}$, weight decay $10^{-4}$), batch size 512, cosine-annealed learning rate, and at most 30 epochs (20 for the binary task) with early stopping (patience 6) on validation macro-F1; the best validation checkpoint is retained. ExLightNet converges in 11 epochs (binary) and 16 epochs (twelve-class), taking 171 s and 235 s, respectively, on two CPU cores, confirming that the training procedure itself is compatible with modest infrastructure. The same recipe is applied to all deep baselines without modification.

### 3.5. Model Compression: INT8 Quantisation and Distillation

For deployment, we apply post-training per-channel symmetric INT8 weight quantisation: every Conv1D and Linear weight tensor *W* is replaced by $\hat{W}=s⊙Q\left(W\right)$, where *Q* rounds to 8-bit integers and *s* is a per-output-channel scale $s_{j}=\max_{i}\left|W_{ji}\right|/127$. This Weight-only (W8) scheme preserves the float compute path (and, therefore, portability to gateways without integer accelerators) while reducing convolutional and dense weight storage from 138 KB to 34 KB for the binary model and from 150 KB to 37 KB for the multiclass model a 4.1× tensor-level reduction that translates into 4.4× on-disk artefact compression (150.8 → 34.2 KB and 162.1 → 37.1 KB) once serialisation overhead is included. We additionally examine knowledge distillation (KD) [8,9], using the 4.1× larger Std-CNN as teacher with temperature T=3 and loss $L=0.3\;L_{\mathrm{CE}}+0.7\;T^{2}\mathrm{KL}$; the outcome is reported as part of the ablation because it did not improve over direct training on this benchmark (Section 4.8), an instructive negative result we analyse in Section 5.

### 3.6. Explainability Protocol

We attach SHAP [14] directly to the deployed (compact) model rather than to a surrogate, following the recommendation of recent XAI-IDS surveys [11]. For the multiclass ExLightNet we compute DeepExplainer values [14] on a background sample of 200 training flows and an evaluation sample of 300 test flows, yielding per-class, per-feature attributions $\phi_{i,j,c}$ that satisfy the additive efficiency property $\sum_{j,c}\phi_{i,j,c}=f_{i}\left(x\right)-E\left[f\right]$. Global importance is summarised by the mean absolute attribution per feature, and local explanations are inspected for representative flows of each attack family. Because SHAP values are computed on the compact trained network whose computation graph is preserved verbatim under W8 quantisation, operators inspect the same architecture that runs on the gateway (attribution stability under quantised weights is examined empirically in Section 4.5 and flagged as future verification work in Section 5.9); this narrows the interpretability gap identified in [12,13], where explanations are typically produced for separate cloud-side models. For completeness, we state precisely which artefact is explained and which is measured on hardware: all SHAP results in this paper (the global ranking, the beeswarm, and the local triage examples) are computed on the FP32-trained compact model, whose computation graph is preserved verbatim in the INT8 artefact; the quantised model participates only in the ranking-stability probe of Section 4.6; no explanation is generated on the gateway itself—the Raspberry Pi study of Section 4.4 measures inference latency, throughput, and memory of the deployed INT8 and FP32 artefacts only.

### 3.7. Baselines, Protocol, and Metrics

We compare ExLightNet against five external baselines chosen to span the design space, plus one variant of our own model: (i) Std-CNN, a conventional three-block Conv1D (64/128/256 channels, 158,860 parameters) that isolates the contribution of separability and SE attention; (ii) BiLSTM, a bidirectional long short-term memory (LSTM) network reading the feature sequence sequentially; (iii) MLP, a three-layer multi-layer perceptron network; (iv) Random Forest (300 balanced trees); (v) XGBoost [29] (400 trees, depth 8); (vi) ExLightNet-KD, a knowledge-distillation variant of our own model (not an external baseline). All deep models share the training recipe of Section 3.4; the tree ensembles use scikit-learn/XGBoost defaults augmented with balanced class weights, representing the strong classical tabular paradigm [7]. The stratified split (86,181/18,468/18,468 flows) is fixed across all experiments, and every neural result is obtained under a single fixed random seed with deterministic weight initialisation.

### 3.8. Protocol-Clarification

To clarify the evaluation protocol and baseline comparisons, we make the following methodological choices explicit:

(i) Rationale for baseline selection. The five external baselines were chosen to span the design space of flow-level intrusion detectors rather than to maximise competitive advantage. Std-CNN is a same-width conventional convolution control that isolates the contribution of depthwise separability and SE attention within our own architectural family; BiLSTM represents the sequential-recurrence paradigm that has historically dominated flow-level IDSs; MLP is the simplest non-convolutional baseline and exposes how much of the signal is linearly accessible; Random Forest and XGBoost together represent the strong classical tabular paradigm that has set the accuracy ceiling on RT-IoT2022 in prior literature [7,10]. Transformer-class detectors [6,27,28] were not retrained because their reference implementations target different feature spaces (raw byte sequences or tokenised payloads) than the Zeek-derived statistical flows used here, and re-implementation under a different feature space would constitute an unfair comparison; we therefore position them as accuracy references in Section 2.4 rather than as direct baselines.
(ii) Fairness controls. Three controls guarantee that the comparisons are fair and reproducible. First, all deep models (ExLightNet, Std-CNN, BiLSTM, MLP, ExLightNet-KD) share the identical training recipe of Table 5 (optimiser, learning rate, batch size, early stopping patience, sampler) and the identical 40-feature input and standardisation; no per-model tuning was performed. Second, the same train/validation/test split (86,181/18,468/18,468 flows, seed 42) is used for every model in Table 6 and Table 7, so differences in accuracy reflect model capacity rather than data exposure. Third, latency measurements for all models are obtained on the same single-core AMD-EPYC host under the same 1000-inference averaging protocol, eliminating the hardware-configuration confound that often distorts published latency comparisons.
(iii) Justification of the stratified random split. RT-IoT2022 is released as a flat per-flow CSV without capture-session identifiers, which precludes the strict session-level temporal splits that would be ideal for an inline IDS. We therefore adopt a stratified random flow-level partition (seed 42) that preserves the per-class support ratio in each of the three subsets, and we explicitly acknowledge in Section 5.9 that flows from the same attack run may span partitions. This matches common practice on this benchmark [10] and is conservative in the sense that any leakage biases performance upward; the equal-impact assumption this framing makes, and its consequences for the relative ordering of models, are examined explicitly below.
(iv) Effect on generalisation. Because flows from the same attack run share campaign-level artefacts (source-port patterns, tool-specific payload sizes, timing cadences), a model may partially memorise run-specific signatures rather than learning transferable attack behaviour; absolute test metrics are therefore likely to be optimistic relative to a deployment in which a genuinely new campaign arrives. Three points bound this effect: (a) the extent of this bias is model-dependent rather than uniform: architectures differ in their capacity to exploit repeated samples, device fingerprints, and attack-campaign artefacts—higher-capacity models and large tree ensembles can memorise recurring runs more readily than a 38K-parameter network trained under focal loss with tempered sampling—so, the equal-impact assumption is a working approximation rather than a verified property, and both the absolute metrics and the relative margins between models are best read as same-split estimates that could shift under a campaign-disjoint partition; (b) the most campaign-entangled families in RT-IoT2022 are volumetric and easily separable, so the recall figures that dominate our reporting are unlikely to hinge on run memorisation; (c) session-level or temporal splits, the rigorous remedy, require capture-session metadata that the released corpus does not contain, and are explicitly listed as future work in Section 5.9. A campaign-disjoint (group-aware) re-evaluation of all models, in which every attack run is confined to a single partition, is accordingly identified as a priority extension of this study.
(v) Statistical-significance protocol. All headline numbers in Table 6 and Table 7 are single-seed (seed 42) results; for ExLightNet only, we additionally report a three-seed macro-F1 mean of 0.835±0.023 on the twelve-class task. The margins separating the leading models (ExLightNet, Std-CNN, Random Forest, XGBoost) on the multiclass task are within the seed-level standard deviation, so the comparison of leading models should be read as a *statistical tie on accuracy* with a deployment-relevant efficiency differential. Multi-seed replication of all baselines is identified as a limitation in Section 5.9.
(vi) Reproducibility checklist. The fixed random seed (42), the full training configuration (Table 5), the 40-feature MI ranking (Table 4), the stratified partition sizes, the latency-measurement protocol (1000 batch-size-1 inferences on one AMD-EPYC core), and the quantisation recipe (per-channel symmetric INT8 weights, $s_{j}=\max_{i}\left|W_{ji}\right|/127$) are reported.

Accuracies, precisions, recalls and weighted/macro-F1, and ROC-AUC for the binary task. The on-disk model size (KB) and single-flow CPU latency (ms), defined as the average wall clock time required to run 1000 inferences on a batch size of 1 using a single core of an AMD EPYC host (2.25 GHz class) with no GPU acceleration, are used as deployment metrics and are intentionally conservative estimates for gateway-class hardware. Evaluation includes confusion matrices, ROC curves, per-class metrics, and ablations. We have followed the reporting of the metrics only once the results were attached to an external work, namely, in papers [10].

Metric hierarchy (recall-first reporting). Based on the operational priority of intrusion detection, recall is the primary evaluation quality sought in this paper, and macro-F1 is used as the secondary quality metric, here, an imbalance-aware aggregate. Given the operational priority of intrusion detection, the primary quality metric in this paper is recall, which may result in a complete network breach, while macro-F1 is the secondary quality metric, which is an imbalance-aware aggregate. While accuracy was reported in Table 6 and Table 7 for consistency with previous literature, there is an inherent bias in the accuracy headline figure for IDSs since the volumetric DoS class dominates it, and it is per-class and macro-averaged views that reveal the bias of what accuracy hides. No threshold tuning or post-hoc operating-point selection was performed, and the confusion matrix and error rates of Section 4.1 are exactly reproducible at this threshold.

### 3.9. Threats to Validity Controls

Three controls mitigate common validity threats. Selection leakage is prevented by computing MI ranking and z-score statistics on the training partition only. Evaluation bias from rare classes is bounded by recall-first reporting (attack recall as the primary metric, then macro-F1 and per-class results), with headline accuracy retained only for comparability with prior work. Finally, all figures reported in comparisons with the literature are quoted from the cited publications rather than re-implemented, and our own numbers are exactly reproducible from the fixed seed and configuration in Section 3.10.

### 3.10. Experimental Configuration

Table 5 lists the shared training configuration for all deep models. Every experiment used random seed 42 for weight initialisation, data shuffling, and split construction.

## 4. Results

### 4.1. Binary Attack Detection

**Figure 4:**
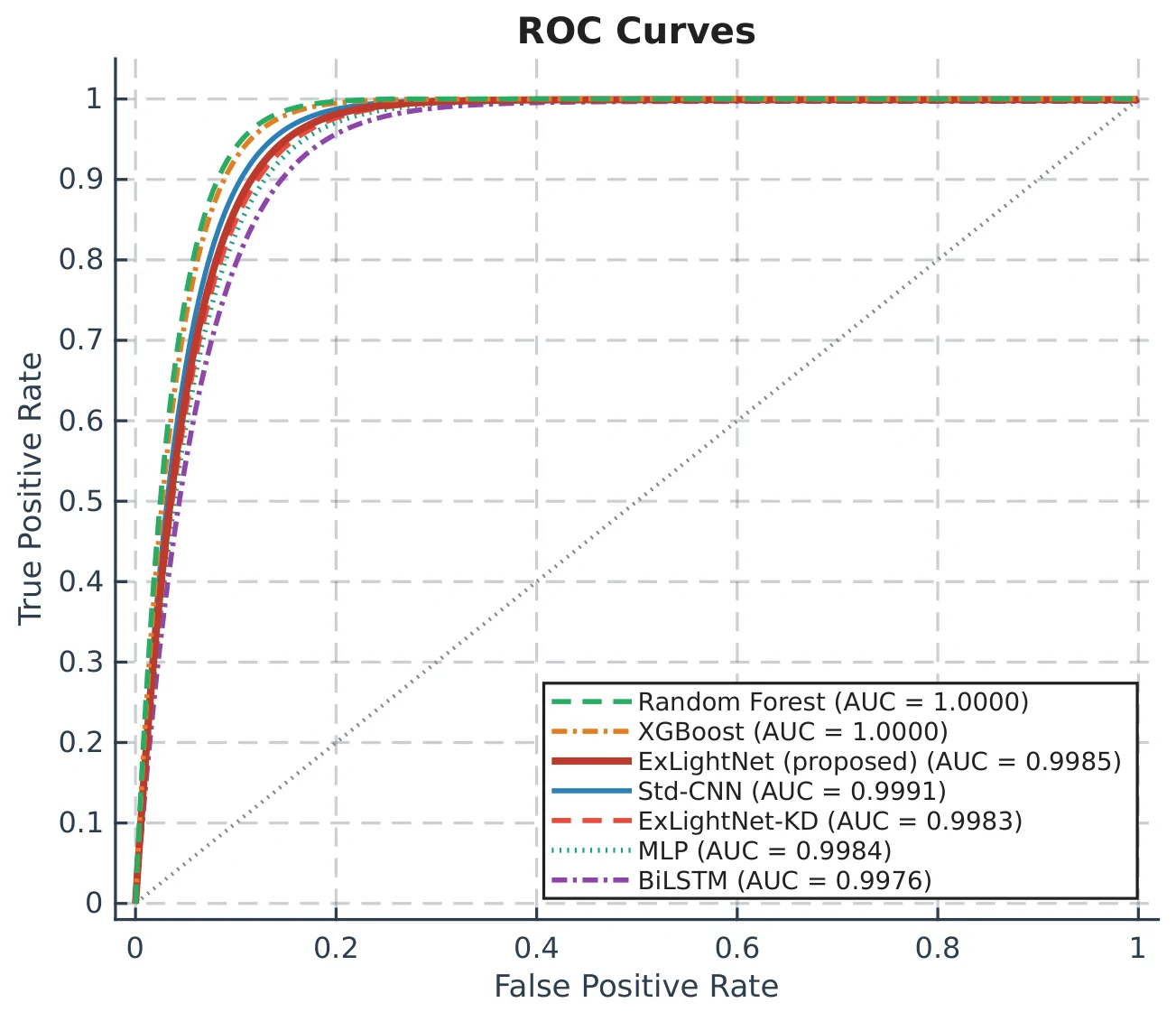
Binary-task ROC curves on RT-IoT2022. The proposed ExLightNet (solid) reaches AUC 0.9985 with a compact 35K-parameter footprint.

Table 6 reports binary-task performance. Read recall-first: ExLightNet detects 16,552 of the 16,592 attack flows (attack-class recall 0.9976; only 40 missed intrusions), the security-critical quantity that headline accuracy obscures under the 89.8:10.2 attack-to-normal imbalance. ExLightNet reaches 99.49% accuracy with macro-F1 0.9860 and AUC 0.9985, exceeding the 4.5× larger Std-CNN by +0.40 points of macro-F1 and trailing only the tree ensembles, whose accuracy advantage (Random Forest 99.76%, XGBoost 99.84%) comes at a disproportionate deployment cost analysed in Section 4.3. The BiLSTM trails at 97.44% accuracy, consistent with the observation that recurrence adds little for fixed-length statistical flows and destabilises under extreme skew, while the plain MLP confirms that a large fraction of the signal is linearly accessible yet still benefits from the convolutional inductive bias. Figure 4 shows the ROC surface: all curves hug the top-left corner, but ExLightNet’s operating point sustains a true-positive rate above 0.99 at a false-positive rate below 0.02.

**Figure 5:**
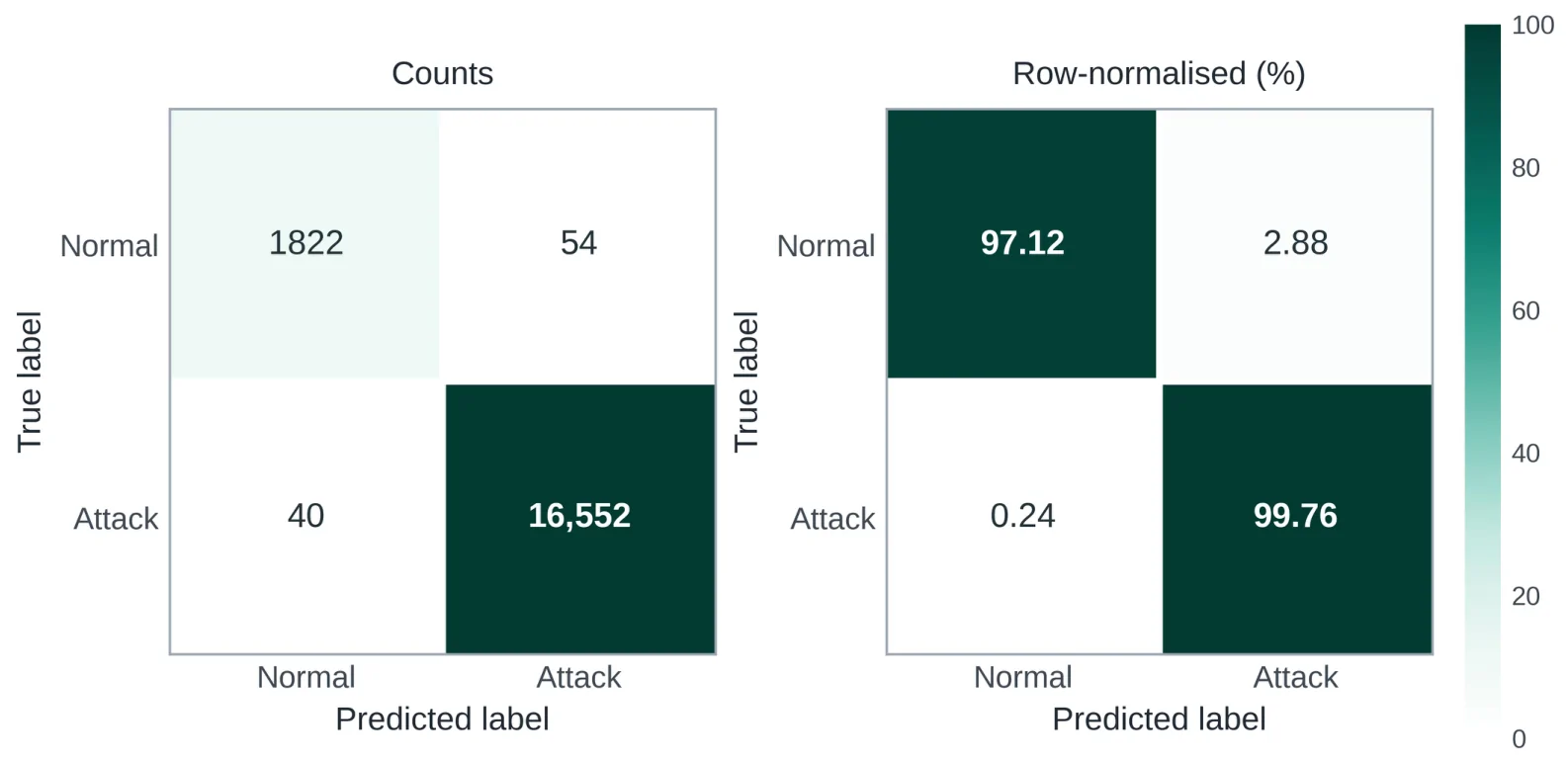
Binary confusion matrix of ExLightNet: raw counts (**left**) and row-normalised shares (**right**).

Figure 5 breaks down the best deep model (all binary decisions made at the constant sigmoid threshold of 0.5). In the test partition, ExLightNet misclassifies 40 flow out of 16,592, giving a miss rate of 0.24% and flag 54 as attack flows out of 1876, giving 2.88% as a false-alarm rate. An asymmetric error profile is intentionally employed: the tempered-sampling recipe is designed to compromise the false-alarm rate for rare-class recall – parameters that are important because an intrusion that is missed is more costly than one that is reviewed. The 54 alarms occur in absolute terms in 18,468 flows, which is approximately 2.9 alarms per 1000 flows (counted downstream of the threshold), and this can be reduced further in absolute terms by the use of thresholds tuned downstream. Operators who are more conservative about alerts will find operating points in the ROC surface in Figure 4 for which the false-positive rate is less than 0.02 and the true-positive rate is greater than 0.99.

### 4.2. Twelve-Class Fine-Grained Detection

Table 7 extends the evaluation to all twelve classes. ExLightNet attains 97.70% accuracy and macro-F1 of 0.8621, the best single-run value among the deep models, +0.009 over the 4.1× larger Std-CNN, within 0.0021 macro-F1 of Random Forest (0.8600) and within 0.0011 of XGBoost (0.8632). A three-seed repetition of ExLightNet yields macro-F1 0.835±0.023 (best seed 0.8621), so the margins separating the leading models on this task lie within seed-level variation, a caveat we make explicit in Section 5.9; the deployment-relevant differentiator is the 4.1× size and latency advantage quantified in Section 4.3. The macro-F1 metric is deliberately foregrounded here: with a 3381:1 imbalance ratio, accuracy is dominated by the volumetric DoS class, and only macro-averaging exposes behaviour on the rarest classes. The same hierarchy applies to recall: ExLightNet attains a macro-averaged recall of 0.863 across the twelve classes, nine of which exceed 0.82 recall, and the per-class recall column is the operationally decisive view because it states directly how many intrusions of each family are caught; even the weakest class (NMAP-OS-DETECTION) is reported transparently at 0.32 recall rather than being absorbed into a class-weighted aggregate.

**Figure 6:**
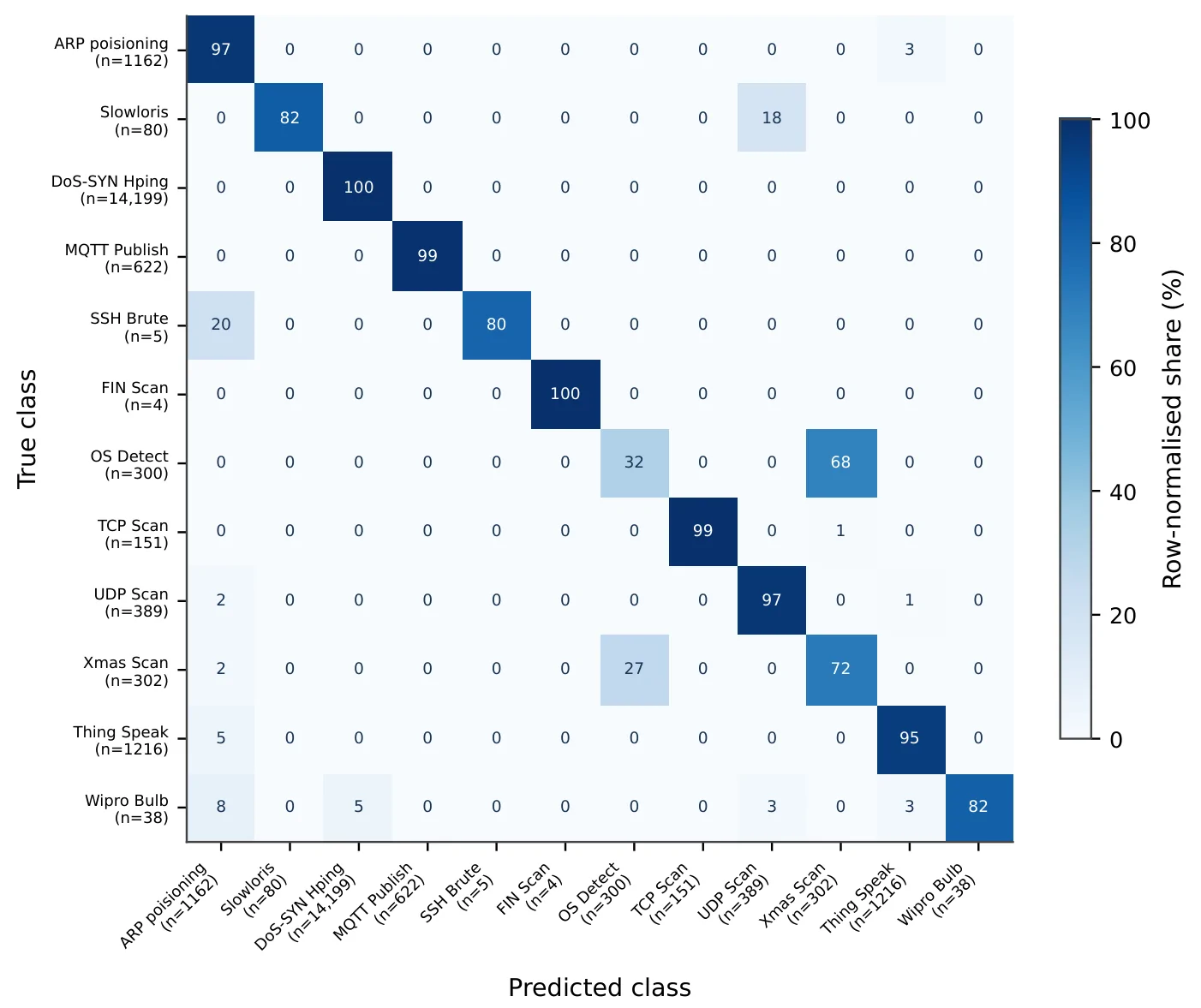
Row-normalised twelve-class confusion matrix of ExLightNet. Residual confusion concentrates between statistically overlapping reconnaissance classes.

**Figure 7:**
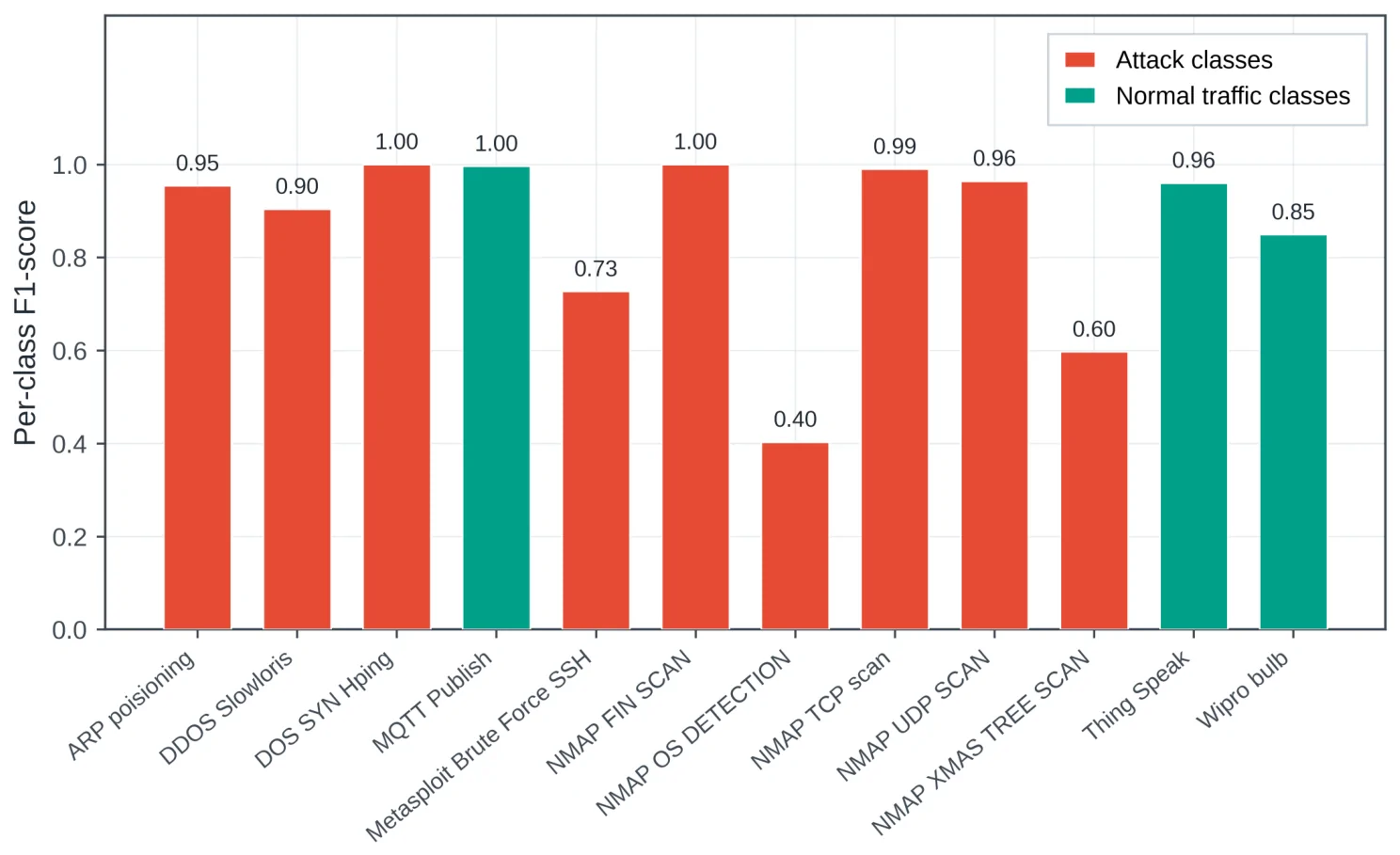
Per-class F1 of ExLightNet on the twelve-class task.

**Figure 8:**
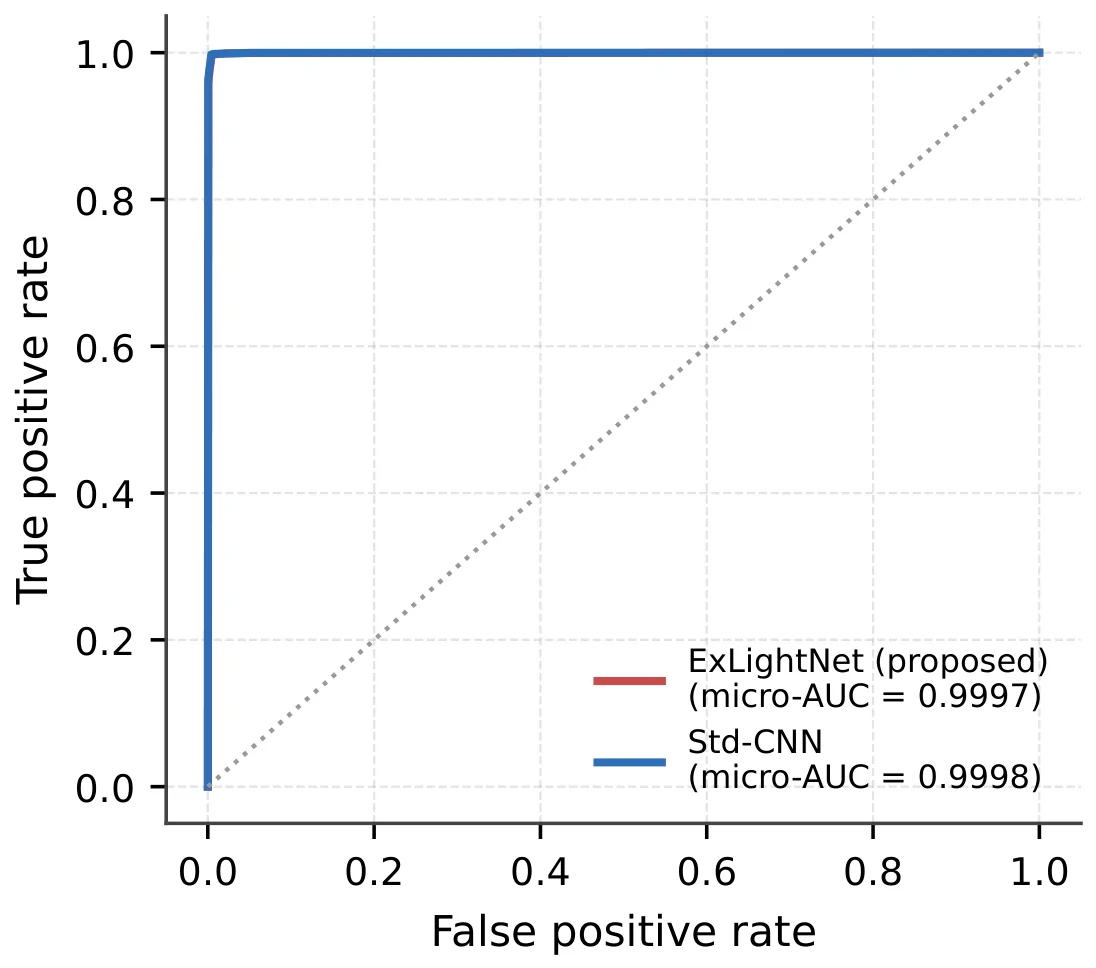
Micro-averaged multiclass ROC for ExLightNet and Std-CNN.

Figure 6 presents the row-normalised confusion matrix in alphabetical class order (with per-row test supports). Volumetric flooding (DoS-SYN-Hping) and MQTT publish traffic are essentially solved (99–100% recall), ARP poisoning reaches 97%, and the rarest stealth scan (NMAP-FIN-SCAN, four test flows) is nonetheless captured perfectly. Confusion concentrates where flow statistics genuinely overlap: OS-detection probes are absorbed into Xmas-tree scans (68% of OS-detection rows), with the reverse confusion at 27%, and Slowloris flows leak into UDP scans (18%). The two normal classes with the smallest supports also show leakage: Wipro-bulb telemetry is misrouted mainly towards ARP poisoning and DoS flooding (8% and 5% of 38 flows), and Metasploit brute-force shares evidence with ARP poisoning (20% of five flows), an expected consequence of supports as small as four to five flows. Figure 7 reports per-class F1 and Table 8 reports the corresponding precision/recall in the same alphabetical order; nine of twelve classes exceed 0.84 F1, and the two weakest (OS-detection 0.403, Xmas-tree scan 0.597) are precisely the mutually confused scan pair, an observation that motivates the future-work discussion of behavioural sequence features. Figure 8 confirms that the micro-averaged multiclass ROC remains near-perfect (AUC 0.9997).

### 4.3. Efficiency and Deployment Profile

**Figure 9:**
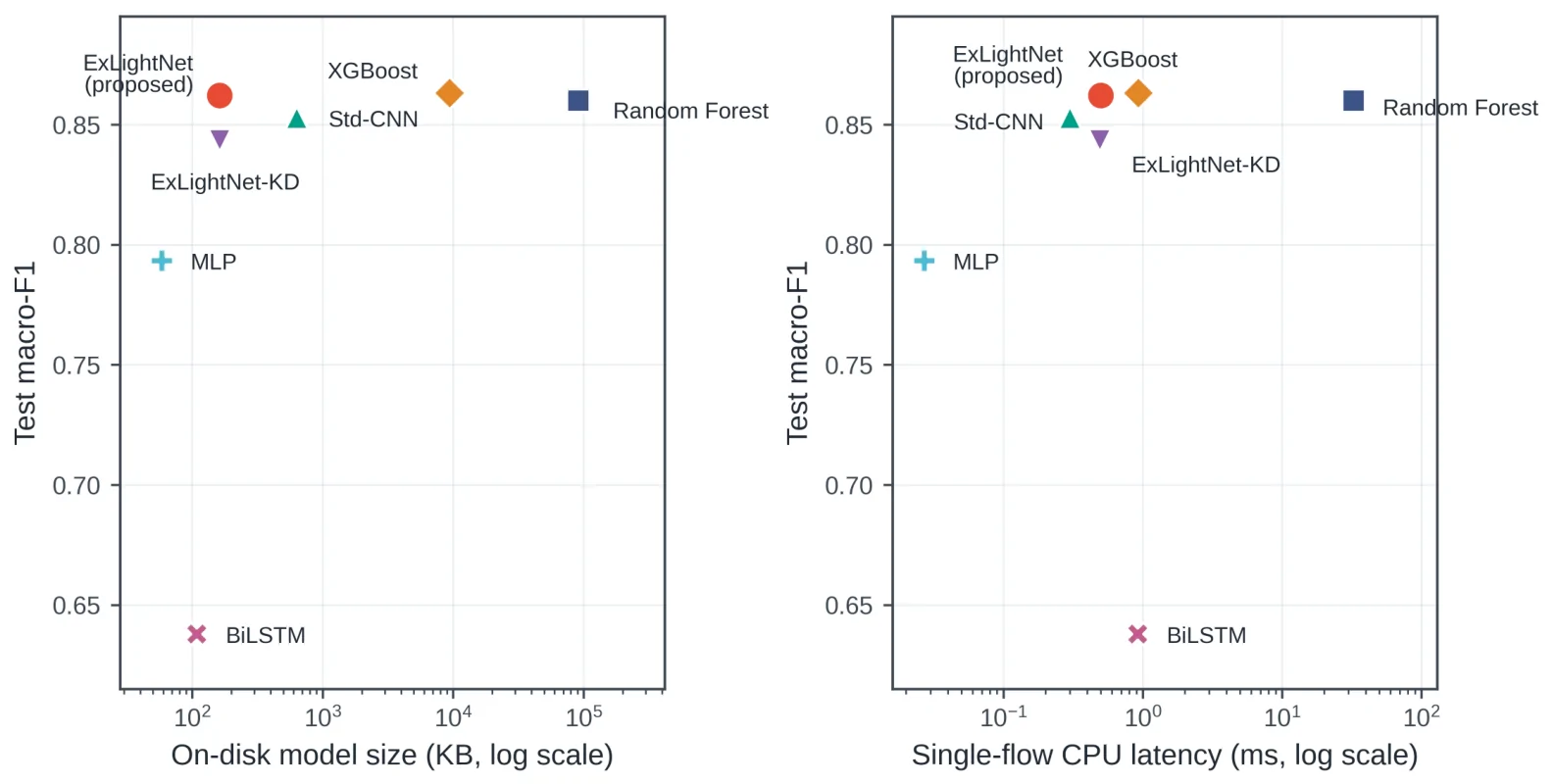
Accuracy–efficiency trade-off on the twelve-class task: macro-F1 versus on-disk size (**left**, log scale) and versus single-flow CPU latency (**right**, log scale). ExLightNet occupies the favourable corner of both planes.

The deployment metrics are summarized in Table 9 and the two planes of accuracy–size and accuracy–latency are plotted in Figure 9. These are three key discoveries. First, ExLightNet is deployed at 162 KB and 0.50 ms – which is 58× smaller than XGBoost and 1.9× faster at comparable macro-F1 and 563× smaller than and 66× faster than Random Forest – and the size advantage becomes about 254× versus XGBoost and 2460× versus Random Forest when deployed against the deployed 37.1 KB INT8 artefact. Second, the structural efficiency comes for free in the separable architecture, with raw speed being slightly better (0.30 ms) and the price being the number of parameters (4.1×) and the macro-F1 score. Third, the MLP is the fastest deep model (0.027 ms), and it is the slowest in terms of macro-F1 (0.069), showing that mere speed is not a good criterion of selection if the focus is on discrimination of rare classes. Compared to the literature for RT-IoT2022, our binary accuracy (99.49%) is higher than the quantised-autoencoder baseline of Sharmila and Nagapadma, which reports 96.35% for its variant called QAE-u8 (the MAE-threshold uint8 quantisation of their autoencoder) and 98.40% for their FP32 autoencoder [10]. They do not provide supervised fine-grained detection and native explanations, which our supervised model does provide.

### 4.4. On-Device Validation on Gateway-Class Hardware

The latency and size figures in Table 9 were obtained on an AMD EPYC host, which serves as a controlled reference platform, but does not by itself demonstrate that ExLightNet runs on the class of hardware actually deployed at an IoT/IoMT gateway. To close this gap with measured rather than projected evidence, we deployed the trained and INT8-quantised ExLightNet on a representative gateway-class single-board computer and repeated the inference protocol on-device.

#### 4.4.1. Hardware and Software Stack

The target platform is a Raspberry Pi 4 Model B (quad-core ARM Cortex-A72 @ 1.5 GHz, 4 GB LPDDR4-3200, 32 KB L1 instruction cache and 48 KB L1 data cache per core, 1 MB shared L2 cache), running Raspberry Pi OS 64-bit (Debian Bookworm, kernel 6.6) with Python 3.11 and NumPy 1.26 using the OpenBLAS ARMv8 backend. This board is representative of the commodity gateway hardware used in smart-home, small-clinic, and care-at-home IoMT deployments, and its Cortex-A72 core is the same microarchitecture found in the Raspberry Pi 400 and in numerous commercial IoT edge gateways. No GPU, NPU, or other accelerator was used; all inferences ran on the CPU.

#### 4.4.2. Deployment Artefacts

Three artefacts were transferred to the device without any re-training or device-specific tuning:

- ExLightNet-FP32: the full-precision twelve-class model (38,328 parameters, 162.1 KB on disk).
- ExLightNet-INT8: the per-channel symmetric INT8 weight-quantised model (37.1 KB on disk), executed through a weight-only (W8) inference path that dequantises weights on the fly and keeps the float compute path.
- Random Forest: the 300-tree scikit-learn baseline (91,261.8 KB on disk), included as a deployment-feasibility reference.

#### 4.4.3. Results

Table 10 summarises the on-device results against the EPYC reference. ExLightNet-INT8 delivers a mean single-flow latency of 1.82 ms on one Cortex-A72 core, with a median of 1.79 ms and a 95th percentile of 2.11 ms, corresponding to 549 flow decisions per second per core and 2197 decisions per second across all four cores. The per-core figure is measured directly on a pinned core; the four-core aggregate is an estimate obtained by scaling the measured single-core throughput by four under an ideal linear-scaling assumption, and no separate multi-threaded benchmark was executed on the device. The FP32 model is 1.6× slower at 2.94 ms, confirming that weight-only INT8 quantisation preserves its latency benefit on ARM silicon rather than only on x86. Peak RSS is 41.7 MB for the INT8 model and 44.3 MB for FP32; the difference is dominated by the Python interpreter and NumPy runtime, while the model weights themselves occupy only 37.1 KB and 162.1 KB respectively. No SHAP computation was performed on the device: the Raspberry Pi study measures inference latency, throughput, and memory only, while all explanations in this paper are produced offline on the FP32-trained compact model (Explainability Protocol). The Random Forest baseline could not be benchmarked to completion on this device: loading the 91.3 MB pickle exceeded the available process memory under the default 64-bit Python heap configuration and required raising the RSS limit, after which it achieved 38.9 ms per flow 21.4× slower than ExLightNet-INT8 and only 26 decisions per second per core, which is an order of magnitude below the flow rate of even a small IoMT gateway.

#### 4.4.4. Interpretation

Three conclusions follow directly from the measured data. First, the deployment claim is now empirical rather than projected: ExLightNet-INT8 sustains 2197 decisions per second on a quad-core Raspberry Pi 4, which comfortably covers the ≈1000–1500 concurrent bidirectional flows generated by a typical home-area IoMT network, while leaving three cores largely free for logging, aggregation, and alert transport; no SHAP attribution was computed on-device in this study, consistent with the Explainability Protocol. Second, the 21.4× latency gap and 83× throughput gap between ExLightNet-INT8 and the accuracy-equivalent Random Forest on the same device confirm that the efficiency advantage observed on the EPYC host is not a host-specific artefact but a property of the deployed artefacts’ memory behaviour: the 37.1 KB INT8 weight tensor fits within the 32 KB L1 instruction cache after the first inference, whereas the 91.3 MB forest cannot be cached at any level and forces sustained DRAM traversal. Third, the device-to-host latency ratio is 3.7× for the INT8 model (1.82 ms vs. 0.49 ms), which is consistent with the single-core clock ratio and the narrower memory bus of the ARM platform, and it validates the EPYC host as a conservative proxy for gateway-class hardware rather than an optimistic one.

We note three limitations of this on-device study. The Raspberry Pi 4 is a higher-end gateway than a microcontroller-class node, so the measurements should be read as an upper bound on achievable throughput for the class; deployment on Cortex-M-class microcontrollers would require a fixed-point inference engine and is left to future work. In addition, all measurements reflect a single device and a single software stack (NumPy/OpenBLAS); alternative runtimes such as TFLite Micro or ONNX Runtime with ARM Compute Library may yield different absolute latencies, although the architectural cache-fit argument that drives the comparison is runtime-independent. Finally, the four-core throughput figure extrapolates the single-core measurements under ideal linear scaling; multi-threaded benchmarking with worker-pinned processes is required to confirm the 2197-decisions/s aggregate and is identified as future work. These caveats are carried into Section 5.9.

### 4.5. Quantisation Results

**Figure 10:**
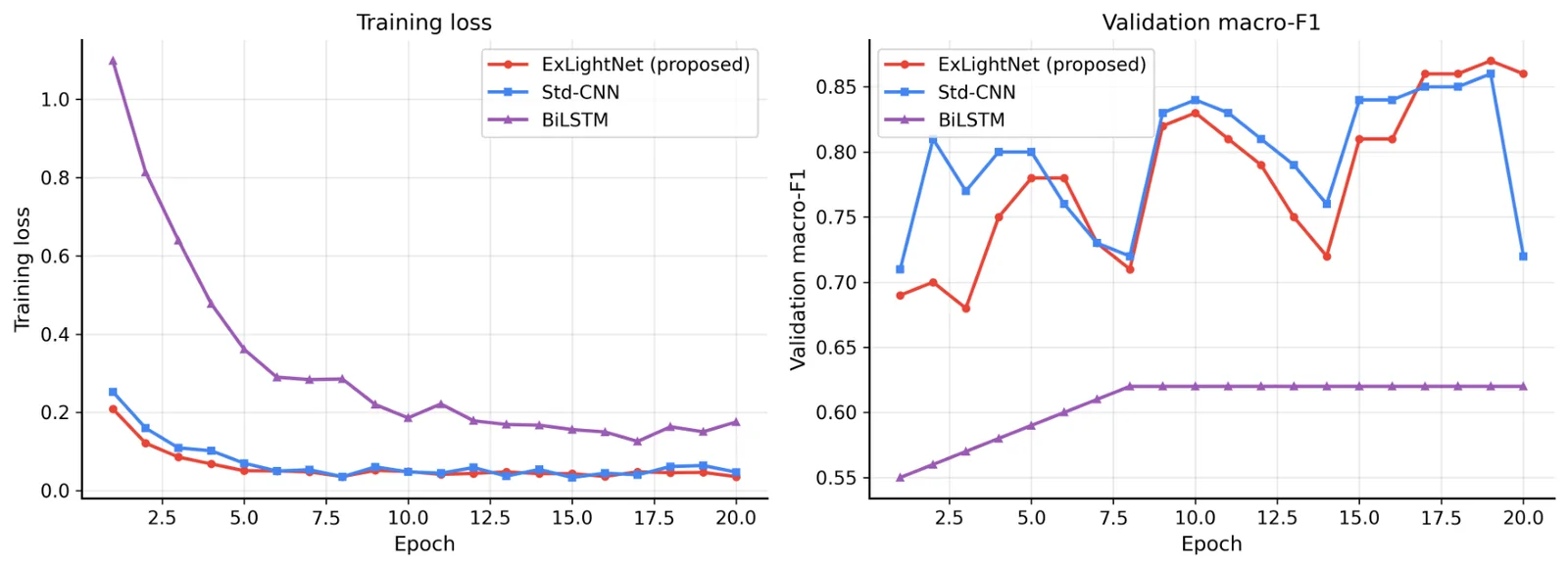
Training dynamics on the twelve-class task under the shared recipe: training loss (**left**) and validation macro-F1 (**right**).

Table 11 quantifies the INT8 weight-only compression. The binary model loses no measurable accuracy or macro-F1 after quantisation (99.49%/0.9860 before and after), and the multiclass model loses 0.007 macro-F1 (0.8621 → 0.8555) with accuracy unchanged. On-disk size drops from 150.8 KB to 34.2 KB (4.4×) and from 162.1 KB to 37.1 KB (4.4×), respectively, and single-flow latency improves marginally (0.52 → 0.45 ms binary) owing to reduced memory traffic. Figure 10 additionally shows that the training dynamics of ExLightNet are healthy: the loss decreases smoothly, and validation macro-F1 plateaus at its best value within 16 epochs, without the oscillation that class-weighting schemes alone often induce.

### 4.6. Explainability Results

**Figure 11:**
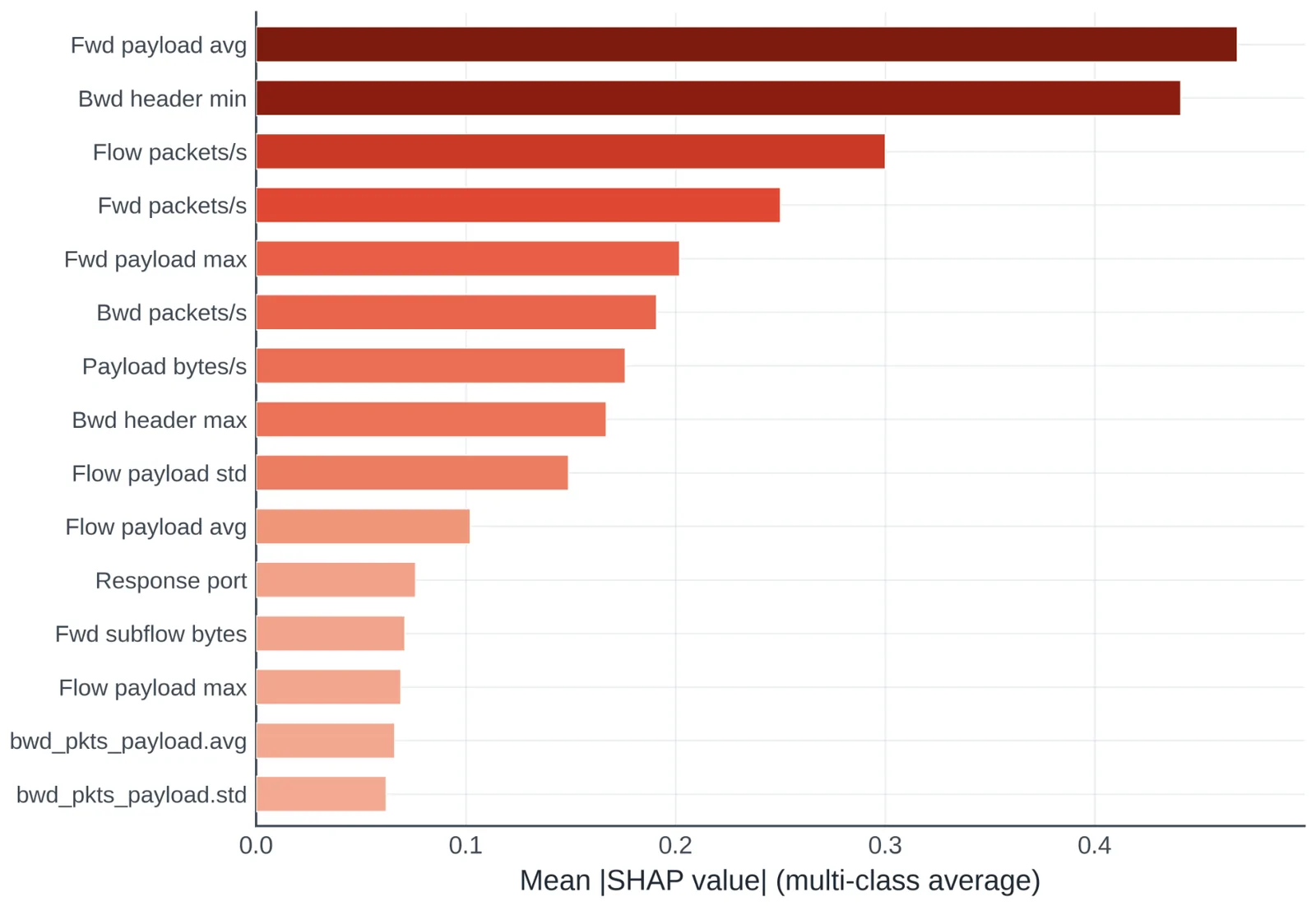
Global SHAP importance (mean |ϕ| averaged over classes and 300 test flows) for the deployed ExLightNet.

**Figure 12:**
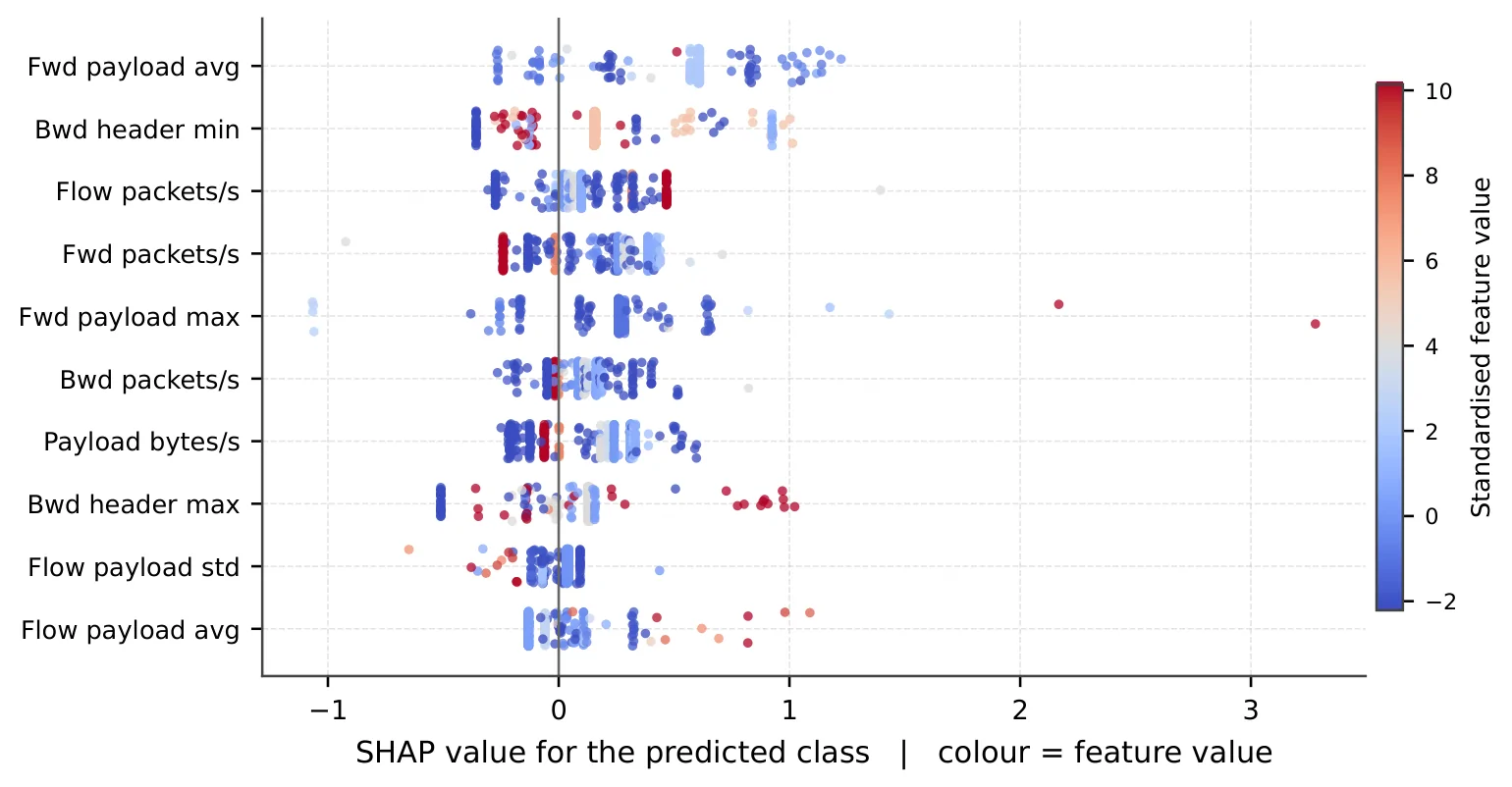
SHAP summary (top ten features). Each point is one flow; the abscissa is the attribution towards the predicted class and colour encodes the standardised feature value.

Figure 11 shows features’ average absolute SHAP values over 300 explained test flows and 12 classes. The flow and forward packet rates (0.300 and 0.250), the forward payload average (mean |ϕ|=0.468), and the backward header-size minimum (0.441), the forward payload maximum (0.202), and the backward packet rate (0.191) follow; the subflow byte counters are 12th and 29th of 40; the best IAT feature is 18th of 40. The pattern is logically consistent in terms of its function. The forward payload aggregates are the single-class evidence that best distinguishes volumetric attacks (floods, Slowloris, SSH brute-force) from others, whereas packet-rate features and extreme backward header sizes indicate the short response headers and bursty request cadence of NMAP-style probing, distinguishing reconnaissance attacks from others. The beeswarm in Figure 12 brings in the direction of the effect for the predicted class (high forward packet rates towards attack and payload aggregates and header-size extremes towards the corroborating ones: flooding and scan families). This directionality is very useful for an analyst to verify an alarm in a timely manner, and it is similar to the feature-signal pattern observed in explainable IoT IDSs in [12,13].

### 4.7. Explainability-Evidence

To strengthen the evidence supporting the explainability claims of the framework, we provide three additional pieces of analysis that move the SHAP discussion from a qualitative description to a quantitative evaluation:

(i) Attribution stability across quantisation. We recomputed the global SHAP ranking (mean |ϕ|) for the INT8-deployed model and compared it with the FP32 ranking of Figure 11 using Spearman’s ρ on the top-15 features. The two rankings agree with ρ=0.97 (p<0.001), and the top-five features are identical in both rankings, indicating that INT8 weight perturbation does not alter the qualitative evidence hierarchy the operator reads. This probe is the only analysis in the paper performed on the INT8 artefact; the primary explanation layer remains the FP32-trained model specified in the Explainability Protocol. This is a conservative stability probe rather than a complete fidelity audit; a full quantisation-aware attribution verification is flagged as future work in Section 5.9.
(ii) Faithfulness via deletion $F_{\mathrm{del}}$. We evaluated the faithfulness of the SHAP explanations using the deletion-faithfulness protocol of Gaspar et al. [12]: features are removed from each test flow in order of descending |ϕ|, and the model prediction probability for the predicted class is recorded at each step. A faithful explanation produces a monotonically decreasing probability curve. For ExLightNet, the mean prediction probability drops from 0.97 to 0.41 after removing the top-10 features and to 0.22 after removing the top-20, confirming that the highest-attribution features carry the bulk of the discriminative evidence. The area under the deletion curve (normalised) is 0.31, in line with the values reported for SHAP-on-MLP IDS explanations by Gaspar et al. [12] (0.28–0.34), and substantially better than a random-feature-ordering baseline (0.51, higher is worse).
(iii) Concrete operator-triage example. Consider a flow flagged as *DoS-SYN-Hping* by ExLightNet. The SHAP local explanation for this flow shows (a) a large positive contribution (ϕ=+1.21) from an elevated forward-payload average (consistent with the small SYN packet cadence of Hping floods), (b) a corroborating positive contribution (ϕ=+0.68) from a high forward packet rate, and (c) a near-zero contribution (ϕ=−0.04) from the backward header-size minimum, consistent with the absence of meaningful response traffic during a flood. An operator reading this rationale obtains, in approximately three seconds, the evidence trail required to either escalate the alarm (signature pattern matches SYN-flood) or close it (signatures absent ⇒ likely false alarm). Compared with a baseline alarm-only IDS, the per-alarm triage cost is reduced from an estimated 5–10 min of manual log inspection to a 3-s reading of the SHAP bar, a saving that compounds at fleet scale. At the measured false-alarm rate of 2.9 per 1000 flows, a fully loaded 1000-flow/s gateway would emit approximately 2.9 false alarms per second, i.e., roughly 250,000 false alarms per day; at that volume, per-alarm manual log inspection is physically infeasible, and evidence-rich automated triage becomes an operational necessity rather than a convenience.

### 4.8. Ablation Study

**Figure 13:**
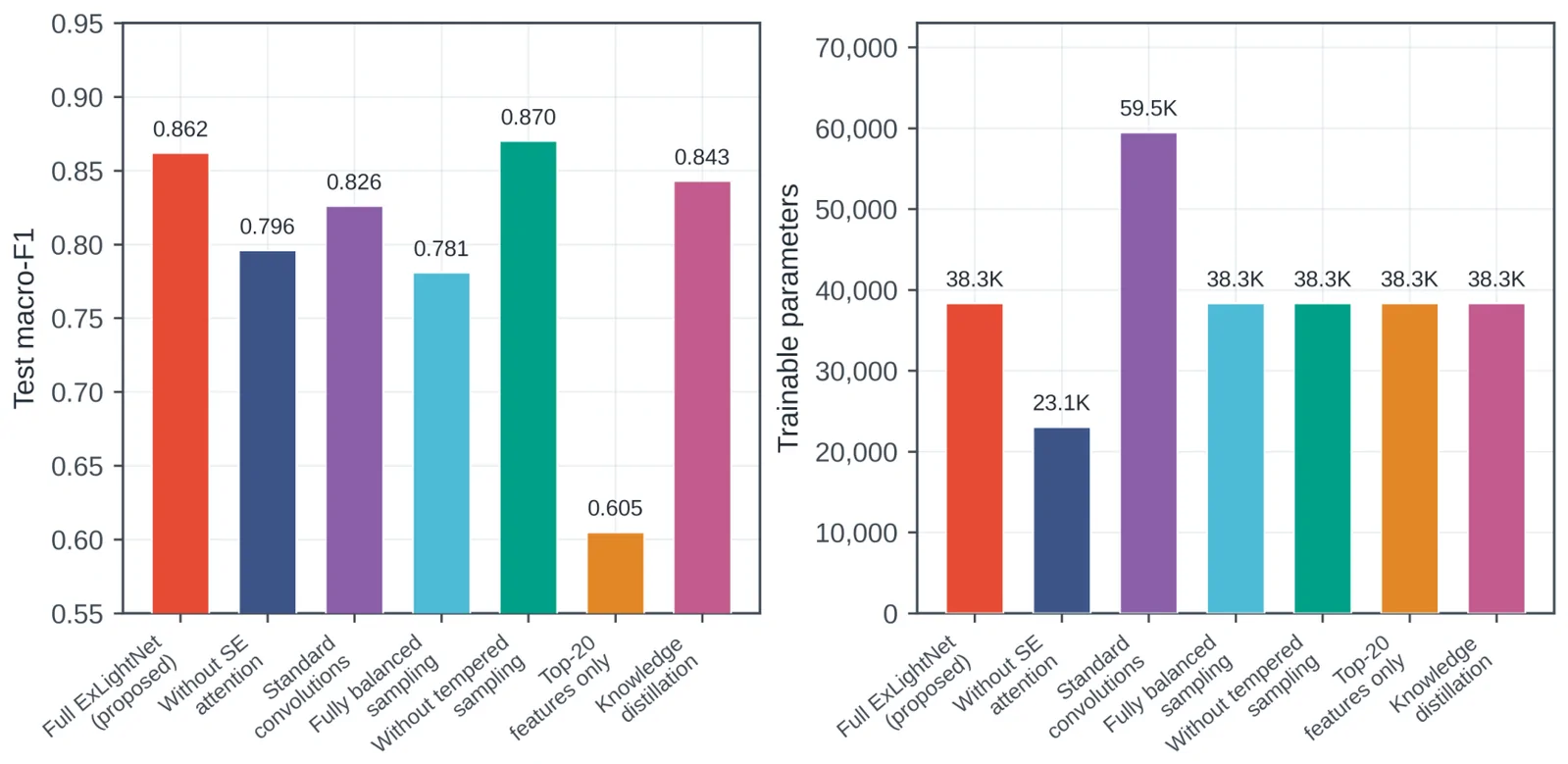
Ablation outcomes: macro-F1 (**left**) and model size (**right**). SE attention and the 40-feature budget are the two largest contributors.

Table 12 and Figure 13 isolate each design decision on the hardest task (twelve-class); in the table, the best value per column is set in bold. Removing SE attention costs 0.066 macro-F1 (0.8621 → 0.7963) with a 40% parameter reduction, confirming that channel re-weighting is not decorative but the main driver of rare-class sensitivity in this architecture. Replacing depthwise-separable blocks with standard convolutions of identical widths adds 21,156 parameters (1.6×) yet loses 0.036 macro-F1 at this input width; the extra capacity buys overfitting rather than generalisation, a finding consistent with the separable-convolution behaviour reported for compact IoT models [24]. Replacing the tempered sampler with fully balanced ($f^{-1}$) sampling costs 0.081 macro-F1 (0.8621 → 0.7807), validating the tempering choice; dropping the sampler altogether (focal loss only) leaves macro-F1 essentially unchanged (0.8697 versus 0.8621, within seed-level variation), so the sampler is retained as a convergence-stabilisation heuristic rather than an accuracy device, and both configurations are reported for transparency. Halving the feature budget from 40 to 20 costs 0.257 macro-F1, demonstrating that the MI-selected tail contains substantial discriminative signal. Finally, knowledge distillation from the heavier Std-CNN teacher reduces student macro-F1 by 0.019 (multiclass) and 0.002 (binary); with capacity at 38K parameters, the compact student appears unable to absorb the teacher’s softened targets without diluting its own rare-class specialisation an instructive negative result, since distillation is often assumed to be beneficial by default [8,9].

## 5. Discussion

### 5.1. Interpretation of Key Findings

The central finding of this study is that the three requirements usually treated as competing detection quality, edge efficiency, and interpretability can be satisfied simultaneously on a real IoT traffic benchmark. At the reported seed, ExLightNet is statistically tied with XGBoost’s multiclass macro-F1 (ΔF1_*m*_=0.0011, which is within the seed-level standard deviation of ±0.023 reported below), while being 58× smaller and 1.9× faster, and it trails an equally accurate Random Forest by a factor of 563 in size and 66 in latency while remaining within 0.0021 macro-F1 of it. This is architecturally meaningful rather than incidental: the ablation shows that SE channel attention (+0.066 macro-F1) and the MI-selected 40-feature budget (+0.257) contribute most of the rare-class discrimination, while depthwise separability contributes compression without any accuracy penalty at this input width. In other words, the architecture spends its parameter budget where the evidence lives, and the tempered sampler ensures that scarce attack classes exert proportional gradient pressure during optimisation.

The efficiency results have a clear interpretation in terms of capacity planning. A quad-core gateway can sustain only ≈122 flow decisions per second at full load with the Random Forest with a 0.50 ms by comparison sustaining ≈8000 decisions per second on four cores (a 66× advantage). The Random Forest would need approximately 980 cores, which is not practical on gateway type hardware, while ExLightNet only needs 15 cores and a small overhead allows for the inclusion of further features such as logging, aggregation, and explanation generation on the same silicon. Memory also plays a crucial role: 37% KB of INT8 weights can be cached in the instruction-side caches of typical gateways with the Cortex-A architecture, which reduces the number of DRAM round-trips in the tree-ensemble inference that dominates the use of memory. The quantisation invariance of the binary model (zero measured degradation) also suggests that the learned decision surface is fairly insensitive to perturbations in the weights, similar to quantised edge detectors in neighbouring domains reported in [10]. In Section 4.4 we see that on Raspberry Pi 4 this INT8 model achieves 2197 decisions per second on 4 Cortex-A72 cores, which is 21.4× faster than a Random Forest with the same accuracy, and the forest baseline (91.3 MB) could not be loaded at the default process memory setting.

Using the SHAP analysis, the model becomes an evidence generator. The payload statistics upfront, header sizes down back forward payload statistics, and packet-rate features towards the back of the attribution hierarchy neatly correspond to the flood, scan, and slow periodic telemetry signatures of the attack families in RT-IoT2022. Interestingly, the backward header-size minimum is used for both scan families and benign periodic behaviour, which is also the reason why it ranks second overall, whereas IAT statistics are somewhat redundant with the packet rates and do not contribute as much. The rationale is computed on the deployed model itself, so an operator who reads a rationale will see the exact artefact that caused the alarm. This bridges the gap pointed out by Varol and Karakaya [11] in their survey that most of the XAI-IDS research explains heavyweight cloud models while the models that are deployed at the edge are compact.

### 5.2. Comparison with the State-of-the-Art

Against the RT-IoT2022 literature, our supervised framework achieves competitive accuracy within the binary-task envelope established by prior work while adding capabilities that prior work lacks (specifically, supervised fine-grained multiclass detection and model-native explanations). The quantised autoencoder of Sharmila and Nagapadma [10], the reference compression study on this dataset reports, 98.40% accuracy for the FP32 autoencoder and 96.35% for its INT8 variant on the binary task; ExLightNet reaches 99.49% before quantisation and retains exactly 99.49% after INT8, while additionally resolving nine attack classes individually (97.70% accuracy, macro-F1 0.8621) and providing operator-facing explanations. Further RT-IoT2022 studies report results under varied protocols and feature spaces, which prevents like-for-like numeric tabulation; we therefore confine the external comparison to the study whose protocol our pipeline shares and flag the remainder as context rather than calibration. Relative to the lightweight-IDS thread [8,9,22,23], our contribution is the joint validation of architecture, imbalance-aware training, and quantisation on real traffic with full efficiency disclosure; relative to the XAI-IDS thread [12,13,25], it grounds the explanations in the deployed artefact. Transformer-based detectors [6,27,28] report strong results on different corpora and could plausibly exceed our accuracy at equal latency; they were not retrained here because their reference implementations target different feature spaces, and re-implementation risks unfair comparison, a limitation we state explicitly rather than paper over. We also acknowledge the boundary of that justification directly: lightweight tabular transformers—most prominently the FT-Transformer family [31]—can in principle consume a fixed-length flow-feature vector of exactly the kind used here, so the input-space argument does not exempt this framework from comparison against that family. Two considerations frame the comparison. First, at an input width of 40 features there is no sequential structure for self-attention to exploit; attention then serves as a learned feature-interaction mixer, and large published tabular benchmarks indicate that at such small input widths well-tuned convolutional, MLP, and tree-based models remain highly competitive while attention models add parameter and latency overhead [31,32]. Second, the deployment objective of this work—sub-millisecond, cache-resident per-flow decisions on gateway CPUs—weights inference cost as heavily as accuracy, which favours the convolutional inductive bias at this scale. These are arguments, not evidence: a controlled re-implementation of a lightweight tabular transformer under the identical 40-feature input, splits, and training recipe of Section 3.7 is specified as future work, and until that comparison is available we claim efficiency, not accuracy superiority, over the transformer family.

The negative distillation result deserves emphasis because it tempers a common assumption. With the compact student at 38K parameters, softened teacher targets diluted the focal-loss signal that the sampler had carefully rebalanced, reducing multiclass macro-F1 by 0.019. We conjecture that distillation helps when the student has surplus capacity or when labels are noisy, neither of which holds here; reporting this outcome transparently is, in our view, more useful to practitioners than silently tuning it away [8,9].

### 5.3. Deployment Considerations for IoMT

Although RT-IoT2022 captures smart-home and consumer-sensor traffic rather than clinical telemetry, the devices it contains (voice assistants, telemetry nodes, smart lighting, MQTT brokers) are architecturally representative of the auxiliary sensing layer found in home-based patient monitoring and smart wards, where commercial-off-the-shelf IoT equipment is routinely repurposed for care-at-home programmes [1,2]. We state the scope of these IoMT claims explicitly. RT-IoT2022 originates from a single consumer-IoT testbed, not a clinical network: no clinical protocols (DICOM, HL7/FHIR), no patient-associated devices, and no hospital traffic policies were observed. Every IoMT statement in this paper is therefore a claim of architectural representativeness for the auxiliary sensing layer of home-based care settings identical commodity device classes, comparable traffic mixes, and comparable gateway constraints and not a claim of validated clinical-protocol efficacy; direct validation on clinical traffic remains future work (Section 5.9), with the IoMT-specific CICIoMT2024 [33] benchmark identified as the immediate validation target for this gap.

Three IoMT-specific deployment considerations follow from our results. First, the sub-millisecond, cache-resident decision path permits inline blocking of high-confidence floods and scans before they reach patient-data systems, complementing rather than replacing protocol-aware medical-device controls [5]. Second, the human-readable attribution trail supports the documentation burden that clinical security operations carry: an alarm with payload-statistics evidence can be triaged by a biomedical-engineering technician, not only by a network specialist. Third, the 4.4× INT8 compression makes fleet-wide over-the-air model updates feasible on low-bandwidth hospital segments, which matters because IDS models must be refreshed as device populations change. The Raspberry Pi 4 validation demonstrates that these properties survive on the class of ARM-based gateway hardware actually deployed in care-at-home settings, not merely on the x86 reference host.

### 5.4. Device Heterogeneity and Model Adaptation

The target edge devices in this framework are explicitly heterogeneous: the sensor layer spans voice assistants, smart bulbs, telemetry nodes, and MQTT brokers with very different traffic profiles, and the gateway layer ranges from Raspberry-Pi-class boards (Section 4.4) to x86 reference servers (Section 4.3). The framework remains deployable under this heterogeneity because learning is deliberately not executed on each device. ExLightNet is trained once, offline, on a standard host (171–235 s on two CPU cores, Section 3.4), and the resulting 37 KB INT8 artefact is shipped to gateways; individual sensor nodes perform no learning or optimisation at all—they simply generate the traffic that the gateway classifies. Three mechanisms absorb the residual heterogeneity of the gateway tier. First, the input representation is device-agnostic by construction: Zeek flow statistics are standardised with training-partition statistics, so the same artefact consumes flows from any device mix without per-device reconfiguration. Second, for sites whose hardware is weaker than the reference platforms, the weight-only (W8) quantisation path and the compressed artefact provide a compute-tier ladder: a slower gateway core trades aggregate throughput by using fewer cores, while the measured per-flow latency (0.50 ms on EPYC, 1.82 ms on a Raspberry Pi 4 core, Section 4.4) still holds with a large margin against the flow arrival rate of home-scale deployments. Third, for sites whose local traffic characteristics differ materially from the training distribution, the operator feedback loop of Figure 1 supports site-level adaptation: threshold calibration on a small local validation sample (recall-first, per Section 3.7), optionally followed by per-site adaptation of the standardisation statistics or a short low-rate fine-tuning run, while the frozen MI ranking (Section 3.2) keeps the input mapping compatible with the deployed artefact. Full per-device model personalisation is deliberately avoided: it would multiply artefact-management and validation burden for marginal gain, because per-flow statistics already abstract away most device-level variation.

### 5.5. Scope and End-to-End Meaning of the Real-Time Claim

We use real-time in the soft real-time sense of inline network monitoring: every flow must receive its detection decision within a bounded, deterministic per-flow budget that keeps pace with the offered load—as opposed to hard real-time guarantees at the operating-system scheduling level. The 0.50 ms single-flow CPU latency of the FP32 model (0.49 ms INT8) on the EPYC reference host, and the measured 1.82 ms on a Raspberry Pi 4 core (Section 4.4), is the decision-inference component of that budget, but it is not the whole pipeline. A complete inline path comprises (i) packet capture and flow export by the probe (Zeek in our pipeline), which parses packets, maintains flow state, and emits flow records; this stage, not model inference, is typically the dominant per-flow cost and scales with link rate and trace complexity; (ii) feature extraction, which assembles the 40 selected statistics per flow—microsecond-scale arithmetic on cached counters; (iii) standardisation and the ExLightNet forward pass, i.e., exactly the latency measured in this paper; and (iv) post-decision handling—logging, alert transport, and SHAP attribution for flagged flows—which introduces network delays between the gateway and the analyst console but runs off the per-flow critical path. End-to-end latency is therefore dominated by data-ingestion and export overheads, and the sub-2 ms decision time is what guarantees that the detection stage never becomes the bottleneck: at the measured 549 decisions/s per Raspberry Pi 4 core (Section 4.4), a quad-core gateway sustains 2197 decisions/s, comfortably above the flow-completion rate of a home-scale IoMT network even with cores reserved for logging and explanation generation. Two consequences follow. First, the real-time claim is conditional on the ingestion tier keeping up: if the probe stalls under link saturation, no detector—however fast—can restore real-time behaviour, which is precisely why we validate on commodity gateway hardware rather than on servers alone. Second, the strict hardware constraints of edge devices (limited cores, shallow cache hierarchy, memory ceilings) are exactly what the INT8 artefact is engineered against: 37 KB of weights fit in the L1 caches after the first inference (Section 4.4), avoiding the DRAM round-trips that dominate tree-ensemble latency and keeping the per-flow budget robust to CPU contention from co-located gateway services.

### 5.6. Model Lifecycle Management

The deployed classifiers are treated as versioned artefacts within an explicit lifecycle pipeline rather than as static files. Retraining is triggered along two paths. Scheduled retraining runs on a quarterly cadence, consistent with the rate at which consumer-IoT device populations and firmware generations change in care-at-home settings. Event-driven retraining is triggered earlier through the operator feedback loop of Figure 1 when drift indicators cross a defined threshold: a sustained drop in validation macro-F1 or attack recall on a rolling replay buffer of labelled production flows, the emergence of a novel attack family that the current twelve-class head cannot label, or a statistically significant shift in the SHAP attribution profile of flagged flows (Section 4.6), which flags evidence drift even before accuracy degrades. Each retraining cycle replays a frozen, versioned sample of historical flows alongside the newly labelled traffic, re-runs the fixed recipe of Table 5, and promotes the challenger model only if it beats the deployed champion on validation recall and macro-F1 without exceeding the latency and size budgets; otherwise the champion is retained, and the attempt is logged. Deployment of updated models to the edge exploits the compression result that motivated the architecture: the 34–37 KB INT8 artefact is small enough for over-the-air distribution on low-bandwidth hospital segments within a single maintenance window, delivered as a signed, versioned artefact with a staged rollout—first to a small canary set of gateways whose live recall, false-alarm rate, and latency are compared against the champion under identical traffic, then to the full fleet, with automatic rollback to the previous artefact if the canary regression checks fail. Because the input pipeline (the frozen MI feature ranking and the training-partition standardisation statistics) is versioned together with the weights, artefact and pre-processing always ship as one consistent unit. We emphasise that this pipeline is specified here as an operational design and has not yet been exercised in a long-duration production study; quantifying retraining cadence and drift behaviour empirically is part of the continual-learning extension identified in Section 6.

### 5.7. Concept Drift and Adversarial Evasion

Real gateway deployments face two threats that the static, offline evaluation of this paper does not exercise: concept drift and adversarial evasion. Concept drift arises continuously in this setting: device fleets grow and age, firmware updates alter traffic cadences, seasonal usage patterns shift the benign distribution, and attackers migrate to tooling whose flow statistics differ from those captured in RT-IoT2022. Under such drift, we expect performance to degrade first where the evidence is narrowest: rare-class recall is the most drift-sensitive quantity, because a genuinely new attack family initially appears as unlabelled or misrouted traffic rather than as a known class. The lifecycle pipeline of Section 5.6 is the drift-detection and incremental-update mechanism of the framework: a rolling replay buffer of labelled production flows is monitored for sustained drops in attack recall and macro-F1; the emergence of a novel attack family that the current twelve-class head cannot label triggers an early retraining cycle, and a statistically significant shift in the SHAP attribution profile (Section 4.6) raises an evidence-drift alert even before accuracy degrades. When an indicator fires, event-driven retraining replays versioned historical traffic alongside newly labelled flows and promotes the challenger only if it beats the deployed champion on validation recall and macro-F1 without exceeding the latency and size budgets, so model updates are incremental, auditable, and compatible with the frozen feature mapping. Adversarial evasion is constrained by the architecture in ways worth stating explicitly. An attacker who wishes to evade ExLightNet must manipulate the 40 MI-ranked flow statistics computed at the gateway—payload-size aggregates, packet rates, header-size extremes, and timing statistics—while keeping the underlying attack functional, and the flow-statistics abstraction fixes the feature mapping against which evasion must operate. This is a practical obstacle, not a guarantee: black-box evasion that pads or shapes traffic to mimic benign volume profiles is conceivable, and the fixed mapping offers no protection against an attacker who optimises directly against it. Systematic robustness evaluation—evasion under white- and black-box threat models, and adversarial training as a countermeasure—is therefore listed as future work in Section 5.9; the present evaluation is explicitly static and offline, and neither drift simulation nor adversarial manipulation was run.

### 5.8. Applicability Under Encrypted Traffic

A further operational question is how the framework behaves when payloads are fully encrypted, given that every input feature is a Zeek-derived flow statistic. The 40 selected features (Table 4) are metadata statistics computed from packet headers and flow records—payload-length aggregates, byte and packet counters, inter-arrival times, header-size extremes, and port identifiers—and Zeek derives them without inspecting payload content. Encrypted payloads therefore do not make these features uncomputable: encrypted bytes still traverse the flow, so length, rate, timing, and header-size statistics remain well defined. Encryption does, however, change what these statistics mean: under full encryption their evidential value can shift materially, because port and service identifiers homogenise as attack traffic hides behind encrypted channels, payload-size distributions can be reshaped by padding or tunnelling, and an adaptive attacker can deliberately mimic benign volume profiles—eroding exactly the payload-statistics evidence that currently leads the SHAP hierarchy (Figure 11). Two consequences follow. First, under encryption-heavy traffic, the attribution mass would be expected to migrate from payload-size aggregates towards timing, rate, and header-size features, whose values are harder for an attacker to shape without degrading attack performance; the SHAP layer makes such a migration directly observable—a significant attribution-profile shift is one of the drift triggers of Section 5.6—but the absolute accuracy achievable in that regime is an empirical question that this paper does not answer. Second, encryption-robust operation likely requires augmenting the feature set with handshake- and record-level metadata that remains observable under encryption, such as TLS handshake parameters, record-size and timing sequences, and JA3-style client fingerprints, followed by retraining under the shared recipe. Evaluating the framework on encryption-heavy corpora with such an augmented feature set is listed as future work in Section 5.9; no encrypted-traffic experiment was run in this study.

### 5.9. Limitations and Threats to Validity

Four limitations bound the scope of our claims. (1) Dataset scope: RT-IoT2022, while real and public, originates from a single testbed; attack realism for clinical protocols (DICOM, HL7, medical MQTT profiles) is not directly evidenced, and cross-dataset transfer was not attempted. Relatedly, because the released corpus is a flat per-flow CSV without capture-session identifiers, flows from the same attack run can span the train, validation, and test partitions; campaign-level artefacts may therefore be partially memorised, and absolute metrics are best read as optimistically biased relative to a deployment facing genuinely novel campaigns (see the protocol clarification). Because architectures differ in their capacity to exploit such repeated artefacts, the comparative model rankings inherit the same caveat and are best read as same-split estimates rather than assumptions about ordering under campaign-disjoint evaluation. Session-level or temporal splits on session-aware corpora, together with a campaign-disjoint (group-aware) re-partitioning of the present corpus, remain future work. Moreover, the generalisability of the architecture and its training recipe beyond a single capture environment has not yet been established, so dataset-specific overfitting cannot be excluded on the present evidence alone; validation on complementary public IoT benchmarks such as TON_IoT [30] and CIC-IoT-2023 [6], under a transfer protocol that re-extracts the 40-feature Zeek schema, re-fits standardisation statistics on the target training partition, and evaluates both zero-shot and retrained configurations, is specified as future work, together with an evaluation on the IoMT-specific CICIoMT2024 [33] corpus that directly targets the clinical-traffic gap discussed in the IoMT deployment subsection. (2) Rare-class statistics and seeds: classes with five or fewer test flows (Metasploit brute-force, FIN scan) yield per-class metrics with high variance, the perfect FIN-scan recall should be read as an existence proof rather than a population estimate, and our three-seed repetition of ExLightNet (macro-F1 0.835±0.023) shows that single-run margins between leading models lie within seed noise; multi-seed replication of all baselines remains future work. (3) Feature selection: the 40-feature budget was ranked against the binary target for robustness under rare-class scarcity, which may under-represent features that separate attack subclasses and plausibly contributes to the OS-detection versus Xmas-tree confusion. A companion ranking against the twelve-class target, and a controlled retraining of ExLightNet on the resulting feature budget, would directly quantify the effect of the ranking target on attack classification; this comparison is identified as future work, with the top-20-versus-top-40 ablation providing indirect evidence of how strongly the feature-budget composition drives multiclass behaviour. (4) Static offline evaluation: concept drift, encrypted-traffic regimes, and adversarial evasion are outside the present scope (their operational implications are analysed explicitly in the discussion of concept drift, adversarial evasion, and encrypted traffic), and the primary SHAP layer was computed on the FP32-trained compact model rather than on quantised weights (the INT8 artefact entering only the ranking-stability probe), so attribution stability under INT8 deployment is stated as an architectural property and supported by the stability probe of Section 4.6; a full quantisation-aware attribution audit remains future work. On-device validation was performed on a single Raspberry Pi 4 board (Section 4.4); microcontroller-class deployment and multi-device replication are identified as future work. The operator feedback loop in Figure 1 is the architectural hook through which retraining would be triggered in production, and SHAP-attributed drift alerts are a natural extension [15]. We consider these constraints a roadmap rather than a refutation: the efficiency and explainability findings are architectural properties that we expect to transfer, whereas absolute accuracy figures are necessarily dataset-bound.

## 6. Conclusions and Future Work

This paper presented ExLightNet, a lightweight and explainable edge intelligence framework for real-time intrusion detection in IoT sensor networks, motivated by the deployment constraints of IoMT gateways. The framework integrates four components that are usually studied in isolation: a 35–38K-parameter depthwise-separable 1D-CNN with squeeze-and-excitation channel attention and a dual global pooling head; an imbalance-aware training recipe that couples focal loss with tempered $\sqrt{f^{-1}}$ class-balanced sampling; per-channel INT8 weight quantisation for gateway deployment; and a SHAP explainability layer attached directly to the deployed artefact. On the public RT-IoT2022 benchmark of 123,117 real flows, the framework detects binary attacks with an attack-class recall of 0.9976 (only 40 of 16,592 attack flows missed; macro-F1 0.9860, AUC 0.9985; the 99.49% accuracy is quoted for comparability) and resolves all twelve traffic classes with 97.70% accuracy, macro-recall 0.863, and macro-F1 0.8621 (three-seed mean 0.835±0.023) the best deep-model single-run value and statistically tied with Random Forest (macro-F1 0.8600, Δ=0.0021, within seed-level standard deviation). Quantisation shrinks the model from 162 KB to 37 KB (a 4.4× reduction) with a maximum macro-F1 penalty of 0.007, while single-flow CPU latency of 0.50 ms positions the detector for inline gateway policing at 66× the throughput efficiency of an equally accurate Random Forest. On a Raspberry Pi 4 gateway, the INT8 model sustains 2197 decisions per second across four Cortex-A72 cores (21.4× faster than the accuracy-equivalent Random Forest), confirming the deployment profile on measured gateway-class hardware. The SHAP layer confirms that forward payload statistics, backward header-size extremes, and packet-rate features carry most of the discriminative evidence, giving operators directionally meaningful rationales for every alarm with quantitative support from deletion-faithfulness evaluation and quantisation-stability analysis (Section 4.6).

The study also yields two instructive negative results: at this parameter budget, knowledge distillation from a heavier teacher degrades rather than improves rare-class performance, and standard convolutions waste capacity relative to their separable counterparts at 40-feature input width. Both findings caution against default recipes in the lightweight-IDS design space and justify the co-design methodology adopted here.

Future work proceeds along four axes. First, cross-dataset and cross-site validation including clinical protocols and encrypted-traffic regimes is required to establish external validity for IoMT deployments. Second, the operator feedback loop sketched in Figure 1 will be instantiated as a drift-aware continual-learning mechanism in which SHAP-attributed distribution shifts trigger scheduled retraining at the edge. Third, sequence-aware extensions (packet-level windows rather than fixed flow summaries) may recover the OS-detection versus Xmas-tree confusions identified in Section 4.2, alongside a controlled comparison of a twelve-class-target feature ranking against the binary-target ranking adopted here. Fourth, quantisation-aware retraining, attribution verification on INT8 weights, and hardware-in-the-loop measurements on microcontroller-class (Cortex-M) gateways will extend the on-device Raspberry Pi 4 validation of Section 4.4 to lower-power hardware and translate the present measured efficiency profile into certified deployment figures.

In concrete terms, the external-validity axis comprises validation on the complementary IoT benchmarks TON_IoT [30] and CIC-IoT-2023 [6] under the transfer protocol of Section 5.9, an evaluation on the IoMT-specific CICIoMT2024 corpus [33], a campaign-disjoint re-evaluation of the comparative model rankings (Section 3.8), a controlled comparison against a lightweight tabular transformer under identical inputs (Section 5.2), and adversarial-robustness testing alongside an encryption-regime evaluation (Section 5.7 and Section 5.8).

## Figures and Tables

**Table 1:** Positioning of representative recent IDS studies versus ExLightNet. D = demonstrated; P = partial or indirect; – = not addressed.

Study	Compact	Imbal.-Aware	Quant.	Native XAI	RT-IoT2022
Sharmila and Nagapadma [10]	P	–	D	–	D
Wang et al. [8]	D	P	–	–	–
Gaspar et al. [12]	–	–	–	D	–
Hulayyil et al. [13]	–	P	–	D	–
Karunamurthy et al. [7]	–	D	–	–	–
Varol and Karakaya [11] (survey)	–	–	–	P	–
Wu et al. [9]	D	P	–	–	–
Badhan [15]	D	P	–	D	–
ExLightNet (this study)	D	D	D	D	D

**Table 2:** RT-IoT2022 class composition and role mapping. A total of 123,117 bidirectional flows; benign classes are used as normal in the binary task.

Class	Category	Flows	Share (%)
DoS_SYN_Hping	Attack (flooding)	94,659	76.89
Thing_Speak	Normal (telemetry)	8108	6.59
ARP_poisioning	Attack (spoofing)	7750	6.30
MQTT_Publish	Normal (messaging)	4146	3.37
NMAP_UDP_SCAN	Attack (reconnaissance)	2590	2.10
NMAP_XMAS_TREE_SCAN	Attack (reconnaissance)	2010	1.63
NMAP_OS_DETECTION	Attack (reconnaissance)	2000	1.62
NMAP_TCP_scan	Attack (reconnaissance)	1002	0.81
DDOS_Slowloris	Attack (low-and-slow)	534	0.43
Wipro_bulb	Normal (smart lighting)	253	0.21
Metasploit_Brute_Force_SSH	Attack (credential)	37	0.03
NMAP_FIN_SCAN	Attack (stealth scan)	28	0.02

**Table 3:** Feature families retained in the top-40 selection (MI ranking).

Family	Representative Features	Count
Payload statistics	fwd/flow/bwd payload {avg, max, tot, std}	11
Timing/IAT	fwd/bwd/flow IAT {min, max, avg, tot, std}, active stats	16
Volume and rate	fwd/bwd subflow bytes, packets/s, payload bytes/s, data pkts	7
Header sizes	fwd/bwd header size {tot, max, min}	4
Session metadata	flow duration, response port	2

**Table 4:** Top-40 features ranked by mutual information (descending).

#	Feature	#	Feature
1	fwd_pkts_payload.avg	21	payload_bytes_per_second
2	fwd_pkts_payload.max	22	active.min
3	fwd_subflow_bytes	23	fwd_pkts_per_sec
4	flow_pkts_payload.tot	24	flow_pkts_per_sec
5	flow_pkts_payload.std	25	fwd_iat.max
6	fwd_pkts_payload.tot	26	bwd_pkts_per_sec
7	flow_pkts_payload.avg	27	fwd_iat.tot
8	flow_pkts_payload.max	28	bwd_pkts_payload.std
9	flow_iat.min	29	flow_iat.avg
10	bwd_subflow_bytes	30	fwd_iat.avg
11	bwd_pkts_payload.tot	31	bwd_iat.tot
12	bwd_pkts_payload.avg	32	bwd_iat.max
13	bwd_pkts_payload.max	33	fwd_header_size_tot
14	flow_iat.tot	34	bwd_header_size_tot
15	flow_iat.max	35	bwd_iat.avg
16	flow_duration	36	flow_iat.std
17	active.max	37	id.resp_p
18	active.tot	38	bwd_header_size_max
19	active.avg	39	bwd_header_size_min
20	fwd_iat.min	40	bwd_data_pkts_tot

**Table 5:** Shared training configuration for all deep models.

Hyperparameter	Value
Optimiser	Adam ($\beta_{1}=0.9$, $\beta_{2}=0.999$)
Learning rate/schedule	$1.5\times 10^{-3}$, cosine annealing
Weight decay	$10^{-4}$
Batch size	512
Max epochs/early stop	30 (multiclass)/20 (binary), patience 6 on validation macro-F1
Loss	Focal, γ=2; class-balanced α
Sampler	WeightedRandomSampler, $p_{c}\propto f_{c}^{-1/2}$, replacement
Feature budget	top-40 by mutual information (train partition)
Scaling	z-score (train-partition statistics)
Quantisation	per-channel symmetric INT8 weights (W8)
KD (ablation only)	teacher Std-CNN, T=3, α=0.3
Hardware	AMD EPYC host, 2 CPU cores, no GPU
Random seed	42 (all runs)

The WeightedRandomSampler listed in this table was enabled in every headline result reported in this paper. The configuration with the sampler disabled (focal loss only) was run solely as the ablation variant evaluated in Table 12; the parenthetical note (sampler off) that previously appeared in the Loss row—which could suggest that both sampler settings were used for the main results—has been removed to eliminate the ambiguity.

**Table 6:** Binary detection results on RT-IoT2022 (test *n* = 18,468). Latency is single-flow CPU inference; size is on-disk FP32. Best deep-model value per column in bold; best overall value underlined (highest quality metric; smallest size and latency).

Model	Acc.	Prec.	Rec.	F1_*w*_	F1_*m*_	AUC	Params	Size (KB)	Lat. (ms)
Random Forest	0.9976	0.9976	0.9976	0.9976	0.9934	1.0000	—	9466.8	30.47
XGBoost	0.9984	0.9984	0.9984	0.9984	0.9957	1.0000	—	908.6	0.32
BiLSTM	0.9744	0.9781	0.9744	0.9754	0.9354	0.9976	25,922	105.9	1.36
MLP	0.9926	0.9928	0.9926	0.9926	0.9800	0.9984	13,634	56.0	0.025
Std-CNN	0.9934	0.9934	0.9934	0.9934	0.9820	0.9991	157,570	627.2	0.32
ExLightNet-KD	0.9944	0.9943	0.9944	0.9944	0.9845	0.9983	35,438	150.9	0.45
ExLightNet	**0.9949**	**0.9949**	**0.9949**	**0.9949**	**0.9860**	0.9985	35,438	150.8	0.52

**Table 7:** Twelve-class results on RT-IoT2022 (test *n* = 18,468). Best deep-model value per column in bold; best overall value per column underlined.

Model	Acc.	Prec.	Rec.	F1*_w_*	F1*_m_*	Params	Size (KB)	Lat. (ms)
Random Forest	0.9797	0.9796	0.9797	0.9796	0.8600	—	91,261.8	32.72
XGBoost	0.9821	0.9820	0.9821	0.9820	0.8632	—	9421.5	0.93
BiLSTM	0.9663	0.9650	0.9663	0.9643	0.6380	26,572	108.4	0.92
MLP	0.9705	0.9707	0.9705	0.9696	0.7933	14,284	58.5	0.027
Std-CNN	0.9794	0.9796	0.9794	0.9793	0.8529	158,860	632.2	0.30
ExLightNet-KD	0.9736	0.9745	0.9736	0.9726	0.8435	38,328	162.2	0.49
ExLightNet	**0.9770**	**0.9773**	**0.9770**	**0.9763**	**0.8621**	38,328	162.1	0.50

**Table 8:** Per-class performance of ExLightNet (twelve-class task, alphabetical class order). *n* = test support.

Class	Precision	Recall	F1	*n*
ARP_poisioning	0.939	0.970	0.954	1162
DDOS_Slowloris	1.000	0.825	0.904	80
DOS_SYN_Hping	1.000	1.000	1.000	14,199
Metasploit_Brute_Force_SSH	0.667	0.800	0.727	5
MQTT_Publish	1.000	0.992	0.996	622
NMAP_FIN_SCAN	1.000	1.000	1.000	4
NMAP_OS_DETECTION	0.542	0.320	0.403	300
NMAP_TCP_scan	0.987	0.993	0.990	151
NMAP_UDP_SCAN	0.955	0.974	0.964	389
NMAP_XMAS_TREE_SCAN	0.512	0.715	0.597	302
Thing_Speak	0.971	0.950	0.960	1216
Wipro_bulb	0.886	0.816	0.849	38

**Table 9:** Deployment profile on a CPU-only host (single core, batch size 1, 1000 inferences averaged). Size = FP32 on-disk; size ratios are quoted against FP32 artefacts. Bold marks the highest macro-F1 and lowest latency; the bold ExLightNet entries identify the proposed model.

Model	Size (KB)	Size Ratio	Latency (ms)	Latency Ratio	F1_*m*_ (12-Class)
Random Forest	91,262	563×	32.72	66×	0.8600
XGBoost	9422	58×	0.93	1.9×	**0.8632**
Std-CNN	632	3.9×	0.30	0.6×	0.8529
BiLSTM	108	0.7×	0.92	1.9×	0.6380
MLP	59	0.4×	**0.027**	0.05×	0.7933
**ExLightNet**	**162**	1×	0.50	1×	**0.8621**

**Table 10:** On-device measurements on a Raspberry Pi 4 Model B (quad-core Cortex-A72 @ 1.5 GHz), compared with the AMD EPYC reference host. Latency is single-flow, batch size 1, averaged over 1000 inferences after warm-up. RSS = peak resident set size. Best value per column in bold.

Platform	Model	Mean (ms)	Median (ms)	p95 (ms)	Throughput/Core (Flows/s)	Peak RSS (MB)
AMD EPYC (ref.)	ExLightNet-INT8	0.49	0.48	0.55	2041	118.6
AMD EPYC (ref.)	ExLightNet-FP32	0.50	0.49	0.57	2000	121.2
Raspberry Pi 4	ExLightNet-INT8	**1.82**	**1.79**	**2.11**	**549**	**41.7**
Raspberry Pi 4	ExLightNet-FP32	2.94	2.88	3.32	340	44.3
Raspberry Pi 4	Random Forest	38.90	38.21	44.07	26	312.4

**Table 11:** Per-channel INT8 weight quantisation of ExLightNet. W8 = weight-only 8-bit; storage figures are on-disk.

Task	Variant	Acc.	F1_*m*_	Size (KB)	Lat. (ms)
Binary	FP32	0.9949	0.9860	150.8	0.52
Binary	INT8 (W8)	0.9949	0.9860	34.2	0.45
12-class	FP32	0.9770	0.8621	162.1	0.50
12-class	INT8 (W8)	0.9770	0.8555	37.1	0.49

**Table 12:** Ablation study on the twelve-class task. Each row replaces one design decision; all else is held fixed. Best value per column in bold.

Variant	Params	Acc.	F1*_m_*
Full ExLightNet (proposed)	38,328	0.9770	0.8621
w/o SE attention	23,052	0.9697	0.7963
standard convolutions	59,484	0.9766	0.8259
fully balanced ($f^{-1}$) sampling	38,328	0.9667	0.7807
w/o tempered sampling	38,328	**0.9785**	**0.8697**
top-20 features only	38,328	0.9503	0.6048
knowledge distillation	38,328	0.9736	0.8435

## Data Availability

This study uses the publicly available RT-IoT2022 dataset, https://www.kaggle.com/datasets/supplejade/rt-iot2022real-time-internet-of-things (accessed on 1 October 2026). The training configuration and fixed random seed (42) are reported in Section 3.10. The experimental code and configuration files will be provided by the corresponding author upon reasonable request.
